# Automatic 3D dense phenotyping provides reliable and accurate shape quantification of the human mandible

**DOI:** 10.1038/s41598-021-88095-w

**Published:** 2021-04-20

**Authors:** Pieter-Jan Verhelst, H. Matthews, L. Verstraete, F. Van der Cruyssen, D. Mulier, T. M. Croonenborghs, O. Da Costa, M. Smeets, S. Fieuws, E. Shaheen, R. Jacobs, P. Claes, C. Politis, H. Peeters

**Affiliations:** 1 OMFS IMPATH Research Group, Department of Imaging and Pathology, Faculty of Medicine, KU Leuven, Leuven, Belgium; 2 Department of Oral and Maxillofacial Surgery, University Hospitals Leuven, Kapucijnenvoer 33, 3000 Leuven, Belgium; 3 Department of Human Genetics, KU Leuven, Leuven, Belgium; 4 Medical Imaging Research Center, University Hospitals Leuven, Leuven, Belgium; 5 Facial Sciences Research Group, Murdoch Children’s Research Institute, Parkville, Australia; 6 Leuven Biostatistics and Statistical Bioinformatics Centre, KU Leuven, Leuven, Belgium; 7 Department of Dental Medicine, Karolinska Institutet, Stockholm, Sweden; 8 Department of Electrical Engineering, ESAT/PSI, KU Leuven, Leuven, Belgium; 9 Department of Human Genetics, University Hospitals Leuven, Leuven, Belgium

**Keywords:** Musculoskeletal system, Oral anatomy, Genetics research, Software, Computational biology and bioinformatics, Image processing, Oral manifestations

## Abstract

Automatic craniomaxillofacial (CMF) three dimensional (3D) dense phenotyping promises quantification of the complete CMF shape compared to the limiting use of sparse landmarks in classical phenotyping. This study assesses the accuracy and reliability of this new approach on the human mandible. Classic and automatic phenotyping techniques were applied on 30 unaltered and 20 operated human mandibles. Seven observers indicated 26 anatomical landmarks on each mandible three times. All mandibles were subjected to three rounds of automatic phenotyping using Meshmonk. The toolbox performed non-rigid surface registration of a template mandibular mesh consisting of 17,415 quasi landmarks on each target mandible and the quasi landmarks corresponding to the 26 anatomical locations of interest were identified. Repeated-measures reliability was assessed using root mean square (RMS) distances of repeated landmark indications to their centroid. Automatic phenotyping showed very low RMS distances confirming excellent repeated-measures reliability. The average Euclidean distance between manual and corresponding automatic landmarks was 1.40 mm for the unaltered and 1.76 mm for the operated sample. Centroid sizes from the automatic and manual shape configurations were highly similar with intraclass correlation coefficients (ICC) of > 0.99. Reproducibility coefficients for centroid size were < 2 mm, accounting for < 1% of the total variability of the centroid size of the mandibles in this sample. ICC’s for the multivariate set of 325 interlandmark distances were all > 0.90 indicating again high similarity between shapes quantified by classic or automatic phenotyping. Combined, these findings established high accuracy and repeated-measures reliability of the automatic approach. 3D dense CMF phenotyping of the human mandible using the Meshmonk toolbox introduces a novel improvement in quantifying CMF shape.

## Introduction

Phenotyping is the complement of genotyping. Just as genotyping extracts the genetic code from DNA, phenotyping extracts quantifiable data from observable characteristics of an organism of interest. Craniomaxillofacial (CMF) phenotyping applies this process to the human face by studying the aspects of facial shape determined by its bony and soft tissue envelope^1,2^. Phenotyping is widely used in biology and anthropology but is also practised in medical and dental specialities. Clinicians assessing craniofacial dysmorphism and diagnosing patients is essentially phenotyping. The results of extensive phenotyping studies on non-pathological humans are also used as a reference for surgical and non-surgical correction of CMF deformation. In these cases, the facial shape is corrected towards ‘normal’ values^3,4^.

In clinical practice, phenotyping still uses rather rudimentary techniques. Facial assessment is based on subjective impressions or multiple univariate measurements between specific predefined anatomical landmarks on the face^5–7^. Classic anthropometric assessment of the soft tissue component has a radiological counterpart named cephalometric assessment. Here, the landmarks are identified on radiological data^8^. The distances and angles between these identified anatomical landmarks are used to quantify facial shape.

There are two important limitations to these techniques. First of all, anthropometric and cephalometric assessment rely on the identification of predefined anatomical landmarks by the assessor. This leaves room for variation and error in the identification of the landmarks: is the assessor capable of identifying the landmarks correctly and if so, are they consistent in locating the landmark? Inconsistency is one of the major pitfalls of manual identification of landmarks^8–10^. Furthermore, only a limited set of landmarks are used for the quantification of shape, underusing the rich nature of the available data in shape^1,11^. This also means that the division between what is normal and abnormal is based on a fraction of the available data.

**Figure 1:**
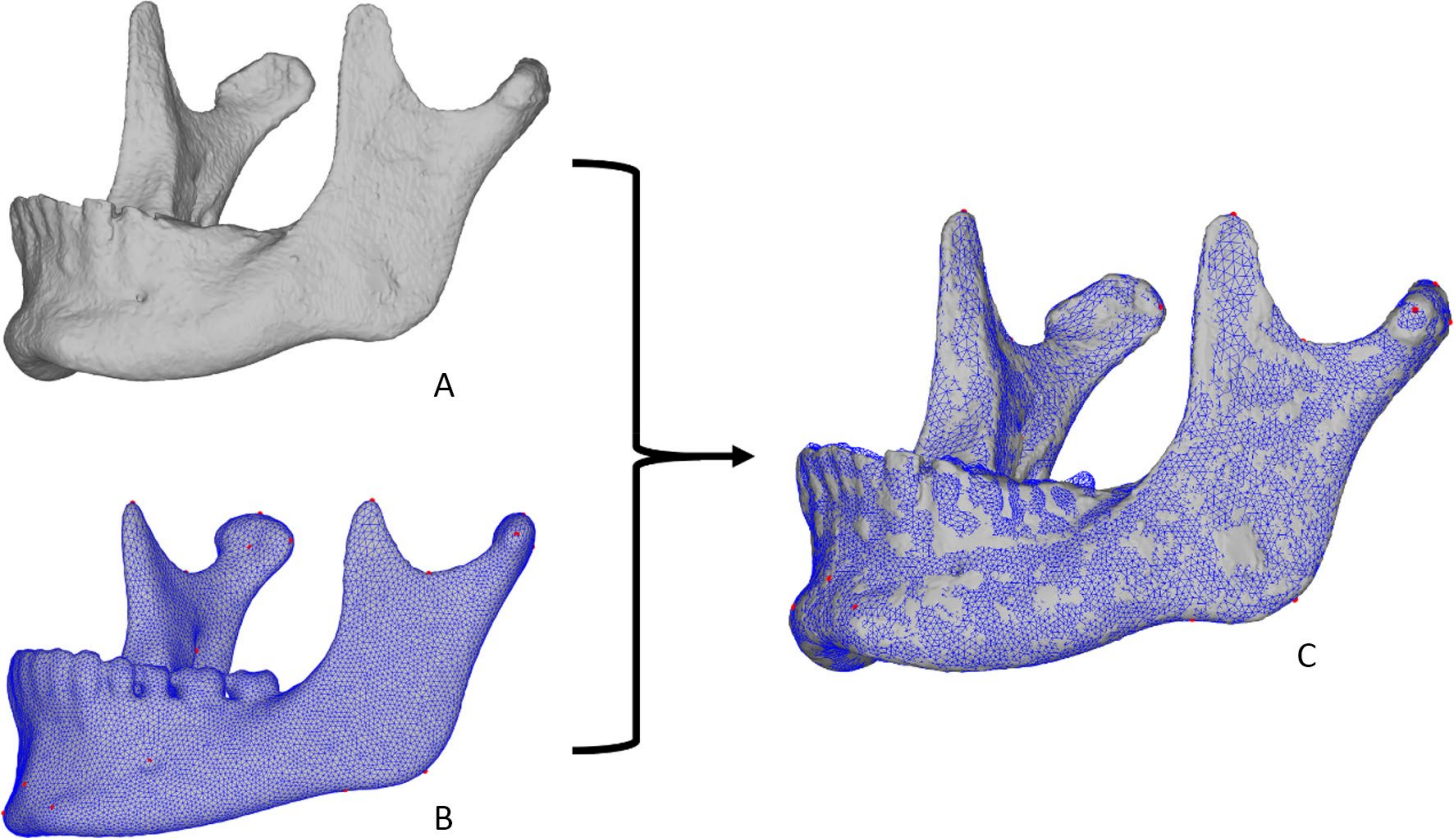
Illustration of the 3D dense CMF phenotyping process. A target 3D mandibular model (**A**) is selected. The Meshmonk toolbox uses a non-rigid surface registration of a template mandibular mesh (**B**) onto the target mandible. The red dots are the 26 anatomical landmarks used in this study and are illustrated more clearly in Fig. 3. The result is a mapped target mandible (**C**) in which all landmarks are always in anatomical correspondence across multiple subjects.

Automatic 3D dense phenotyping is an alternative approach in quantifying facial shape. It requires capturing and constructing a 3D model of a face or bony structure. For the soft tissues this can be done by 3D photography^12^ and for the skeletal structures, segmentation techniques can extract 3D skeletal surface models out of (cone beam) computed tomography ((CB)CT) data^13^. Next, non-rigid surface registration algorithms are used to automatically wrap a dense configuration of thousands of points, a mapping template, across the entire surface of the structure of interest^11,14^. These thousands of points are called quasi-landmarks, and they replace the sparse set of anatomical landmarks used in the classic phenotyping approach. Whenever this technique is applied to a sample of multiple subjects, all quasi-landmarks of the mapping template are dispersed over the structure’s surface, and they are in anatomical correspondence across those multiple subjects (Fig. 1). As an example, quasi-landmark 333 will always be located on the lateral pole of the left mandibular condyle and quasi-landmark 777 will always be located on the center of the right tuberculum mentale. This will be the case for each 3D model of a specific anatomical structure that is subjected to this procedure, no matter if it is derived from different patients or from multiple CBCT scans of the same patient.

3D dense phenotyping was introduced in the early years of 2000^15^ and since then has seen an increase in robustness and its ability to automatically handle large-scale datasets^1,11,16^. The main application of the technique has mostly been the soft tissue facial envelope. This resulted in the development of an open-source automatic 3D dense large-scale phenotyping toolbox called Meshmonk. The toolbox has so far only been tested and validated on facial soft tissue shape where it has proven to be reliable and accurate^11^. The question remains how the same techniques perform when they are applied on the underlying complex bony structures of the face. Proper assessment of the reliability and accuracy of automatic 3D dense CMF phenotyping of 3D models of bony structures is required before it is applied in a clinical or research environment. This study aimed to validate automatic 3D dense CMF phenotyping of the human mandible using a spatially-dense non-rigid surface registration technique. The repeated-measurement (RM) reliability and accuracy will be evaluated with classic phenotyping, using manual landmarks, as a reference. The robustness of the technique will be assessed by applying it on a ‘normal shape’ (unaltered mandibles) and a ‘complex shape’ (operated mandibles) sample.

## Materials and methods

### Study sample

**Figure 2:**
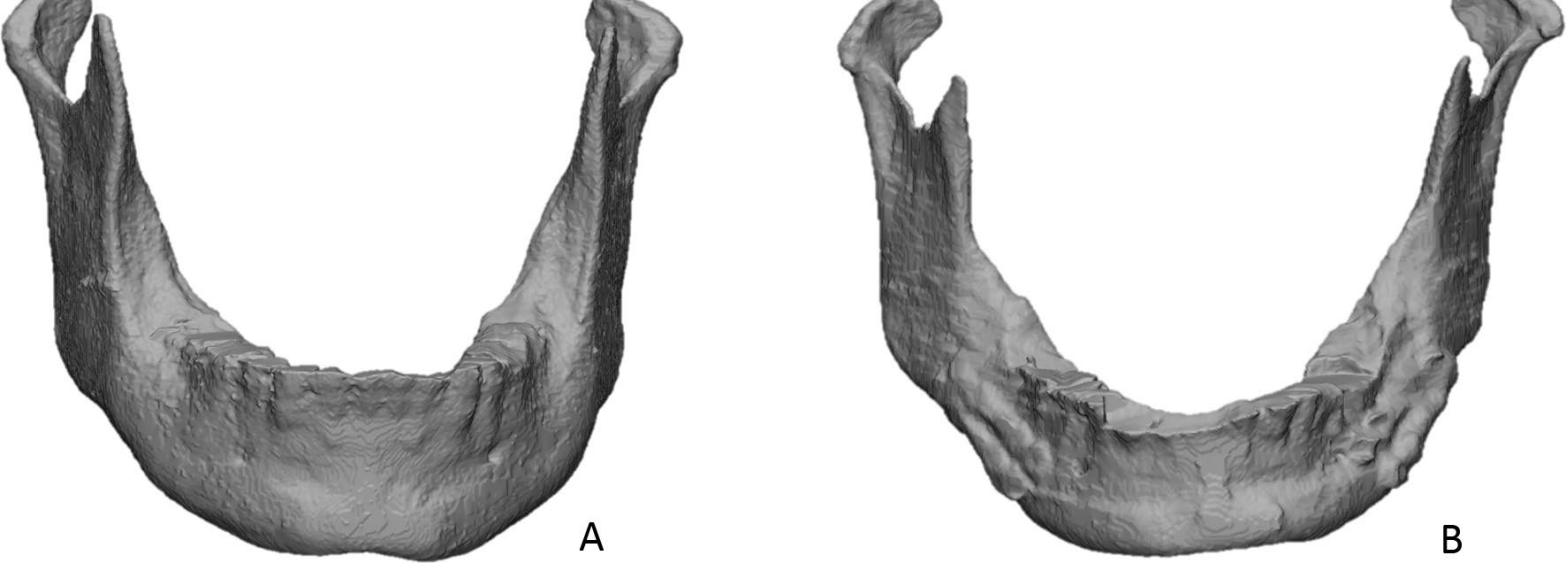
Illustration of mandibles from both samples. The unaltered mandible (**A**) provides a clear-cut anatomical shape. The surgically altered mandible (**B**) has a more irregular outline caused by healed bone cuts and titanium plates.

For this study, we used two samples of human mandibles. The first sample contained 30 anonymized mandibular 3D surface models, constructed out CBCT-scans taken for the virtual planning of orthognathic surgery. This sample was labelled as the unaltered sample as the shape of the mandible is not surgically altered. The second sample contained 20 anonymized operated mandibles that were constructed out of 6-month follow-up CBCT scans. This sample was labelled as the operated sample due to its altered shape in which the teeth bearing part of the mandible is displaced resulting in a more challenging and complex shape for the non-rigid registration technique to process (Fig. 2). All scans were taken with a NewTom Vgi Evo CBCT device (QR Verona, Verona, Italy) with the following imaging parameters FOV 24 × 19 cm, voxel size 0.3 mm^3^, 110 kVp and 4.3 mA. 3D surface models were constructed using a standardised and validated semi-automatic segmentation technique^17^. All scans were taken for clinical purposes and ethical approval to use these scans for research was obtained (LORTHOG Register, Department of Oral and Maxillofacial Surgery, University Hospital Leuven, Belgium, Ethical committee approval UZ Leuven B322201526790). All methods were carried out in accordance with relevant guidelines and regulations and informed consent was obtained from all subjects.

### Phenotyping

**Figure 3:**
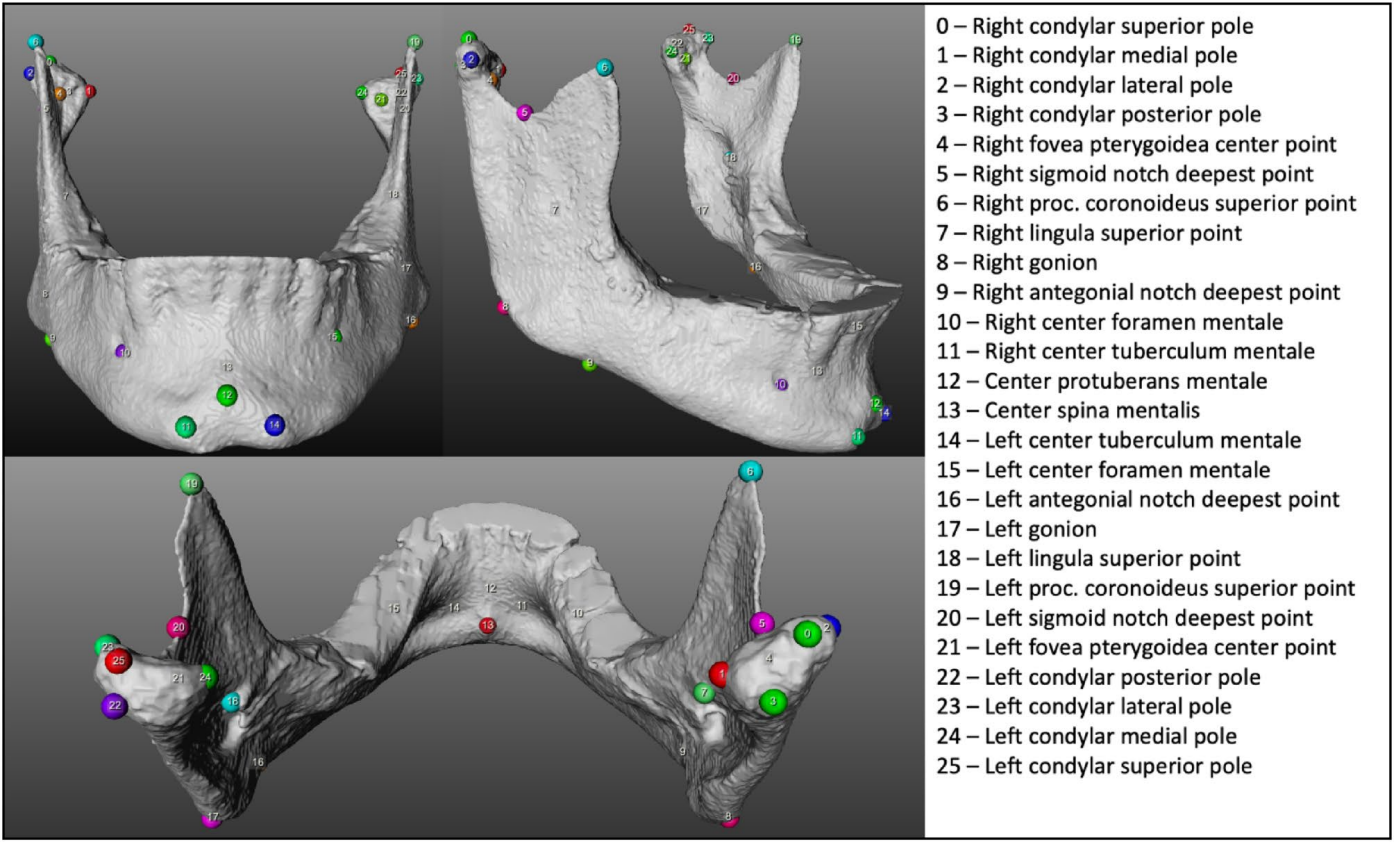
Overview of the 26 manually identified landmarks.

All 3D models of the mandibles were subjected to classic phenotyping by seven oral and maxillofacial residents, well versed in CMF anatomy and cephalometric assessment. After a calibration session where the landmarks of interested and the landmarking tool were introduced, they identified 26 anatomical landmarks on the models using a custom-built network in MeVisLab (available: http://www.mevislab.de/) (Fig. 3). Each resident performed the manual landmarking three times with an interval of 48 h. Automatic phenotyping was performed using a template mandibular mesh in the Meshmonk toolbox. The template mandibular mesh was constructed out of 3D models derived from high resolution CT scanning of 151 dry cadaver mandibles (MANATOMY register, Department of Oral and Maxillofacial Surgery, University Hospital Leuven, Belgium, NH019 2019-09-03). This data was non-rigidly registered with a pre-existing mandibular template derived from cone-beam CT of adolescents^18^. The resulting meshes were co-aligned and scaled to a common size with generalised Procrustes analysis. Averaging the co-ordinates across all 3D models resulted in the template mesh seen in Fig. 1. The Meshmonk toolbox performs a non-rigid surface registration of the template mandibular mesh onto a target mandible after initialisation by a two-step scaled-rigid transformation of the template onto the target as seen in Fig. 1. Firstly, it used five crudely indicated positioning landmarks (left and right lateral condylar poles, left and right gonion and the center of the protuberans mentale) indicated on both template and target to estimate the scaled-rigid transformation. This was further refined using a rigid iterative closest point registration of the template onto the target also implemented in the Meshmonk toolbox. Subsequently, the non-rigid registration is performed which alters the shape of the mapping template to match the shape of the target mandible. The mapping template is a closed mesh consisting of 17,415 quasi-landmarks. The automatic 3D CMF phenotyping procedure is intended to match each of those quasi-landmarks to the corresponding anatomical position on the target mandible.

Before an accuracy assessment could be performed, the 26 anatomical landmarks used in the classic phenotyping approach needed to be identified in the set of 17,415 quasi-landmarks of the mapping template. For each manual landmark (ML) identified on specific mandible by a specific observer, we identified the corresponding automatic landmark (CAL). The mandible of interest was labelled as the target mandible, and the remaining mandibles in the sample functioned as a training set. This leave-one-out approach was preferred to avoid training bias and resulted in a CAL for every ML identified by an observer. After each of the training mandibles had been mapped using Meshmonk, the manually indicated landmarks on the original surface were transferred to the mapped surface. To achieve this, a weighted sum of the three closest points on the mesh (barycentric coordinates) was calculated. These barycentric coordinates were then transformed back to Cartesian coordinates to pinpoint the location of the landmark on the mapping template. As this was done for all training mandibles, all landmark locations were subsequently averaged, resulting in them not always ending up on the surface of the mapping template. This was solved by closest point surface projection in which the landmark was projected on the surface using the shortest distance to that surface and so resulting in the identification of the corresponding quasi-landmark. This method was slightly adapted to establish CAL for the RM reliability assessment as there is direct comparison with the manual method, as there is in the accuracy assessment. The leave-one-out approach was therefore omitted and all manually identified landmarks were used to train the algorithm in finding the 26 CAL among the 17,415 quasi-landmarks on the mapping template, independently for both samples.

### Repeated-measurement reliability assessment

For the validation of automatic 3D dense CMF phenotyping, its RM reliability was compared with the inter- and intra-observer RM reliability of classic phenotyping. Although an automated software is evidently self-consistent, the meshmonk toolbox requires identifying 5 initialisation landmarks which initiates the registration. It is this initialisation phase which still provides some variation in automatic landmarking. Therefore, reliability of the automatic phenotyping was assessed in comparison to classic phenotyping. The root mean square (RMS) distance of a set of indications to the centroid of that set was used as error statistic. The centroid is the mean position of all the points in a specific set of points. RMS distance to the centroid was calculated as the root square of the mean of the squared Euclidean distances of each repeated indication to the centroid of a set of indications. The smaller the RMS distance, the more consistent a (quasi)-landmark was indicated.. Intra-observer reliability of classic phenotyping was assessed for each of the seven observers who produced three sets of 26 landmark indications. The inter-observer reliability of classic phenotyping was tested over the seven observers and used the averaged (n = 3) indications of the 26 landmarks by each observer. For the RM reliability assessment of automatic phenotyping, the coordinate values of the 17,415 quasi-landmarks identified by three rounds of automatic phenotyping were used. However, to be able to make a fair comparison between both methods, RMS distances were also calculated for the 3 sets of CAL, as derived by the three initialisation rounds. Descriptive statistics (mean, standard deviation, minimum and maximum RMS distance) were compared between the classic and automatic method.

### Accuracy assessment

Accuracy assessment was done using the average (n = 3) ML and CAL coordinate values. Three assessment approaches were used. First, for each of the 26 landmarks the Euclidean distance between the MLs and CALs was calculated. This straightforward approach gives a good overview of how far away MLs and CALs are located from each other. Further, we compared shape and size measurements derived from the ML and CAL configurations. The similarity of the centroid sizes of the landmark configurations was assessed^19^. Centroid size is a measure of size in geometric morphometrics and is the square root of the sum of squared distances of all the landmarks of an object from their centroid^20^. A Bland–Altman plot was given to visualize the agreement between centroid sizes averaged over all observers. A linear mixed model with random effects of mandible (n = 30 or n = 20) and observer (n = 7) was used to compute the contributions of mandible, observer and method to the total variance of the centroid sizes. Note that the error variance in this model refers to the contribution of method. Mixed models were fitted using PROC MIXED in SAS version 9.4. (SAS Institute Inc., Cary, NC, USA) using restricted maximum likelihood estimation (REML). These variance components were used to calculate the intra-class correlation coefficient (ICC), the standard error of measurement (SEM) and the reproducibility coefficient (RC), the latter being 2.77 times the standard error of measurement (SEM). The RC is the value below which 95% of the differences between both methods lie. Finally, the full set of inter-landmark distances can be regarded as a very comprehensive representation of the shape of the given landmark configuration^21^. For each landmarked mandible, a set of 325 distances represent the specific shape of that mandible. So, to check if the automatic and classic phenotyping achieved similar shape configurations, a generalisation of the classic ICC for multivariate data was used on these interlandmark distances^22^.

## Results

### Repeated-measures reliability

Table 1 shows the error statistics for evaluating automatic and classic phenotyping RM reliability averaged over all landmarks (26 ML and 26 CAL). Intra- and inter-observer error statistics for each of the 26 MLs are shown in Supplementary Materials 1 and 2. The repeated-measure RMS statistics for the 26 CAL’s are found in Supplementary Material 3. Automatic phenotyping had very low RMS distances of 0.0067 mm (unaltered sample) and 0.0077 mm (operated sample) averaged over the 26 CAL, indicative of high consistency. The condylar region stood out as one of the most reliably mapped regions of the mandible (Supplementary Material 4). Inter- and intra-observer error for classic phenotyping was much higher. The intra-observer averaged (n = 26) RMS distances ranged from 0.75 to 1.17 mm in the unaltered sample and was slightly higher in the operated sample (0.84–1.20 mm). The inter-observer averaged (n = 26) RMS distance was 1.18 mm in the unaltered sample and 1.40 in the operated sample. The principal axes of intra- and inter-observer variation for MLs were calculated as well (Supplementary Materials 5–8). These results showed that some of the condylar landmarks as well as the gonial angle and chin landmarks had been susceptible to high variation when they are manually indicated.

**Table 1:** RMS distances (mm) of repeated landmark indications to the centroid of that set of indications. Averaged over n = 26 corresponding automatic landmarks for automatic phenotyping and n = 26 manual landmarks for classic phenotyping.

	Unaltered sample					Operated sample				
	Mean	95% CI mean	Std	Min	Max	Mean	95% CI mean	Std	Min	Max
Automated	0.0067	0.0043–0.0092	0.0061	0.0019	0.0217	0.0077	0.0045–0.0109	0.0079	0.0012	0.0318
Inter-operator	1.1778	0.9808–1.3748	0.4878	0.4880	2.1179	1.4046	1.1216–1.6876	0.7007	0.4807	3.4909
Intra-operator 1	0.9952	0.846–1.1444	0.3695	0.4767	1.9480	1.1175	0.8482–1.3868	0.6667	0.3282	3.3394
Intra-operator 2	1.0411	0.8751–1.2070	0.4109	0.4214	1.8050	1.0963	0.8677–1.3249	0.5659	0.4584	3.1026
Intra-operator 3	0.9125	0.7582–1.0668	0.3821	0.3348	1.9277	1.0171	0.8323–1.2019	0.4575	0.3196	2.3343
Intra-operator 4	1.1702	0.9858–1.3545	0.4565	0.5609	2.2880	1.1751	0.9038–1.4464	0.6717	0.4617	3.8427
Intra-operator 5	1.0861	0.8791–1.2931	0.5125	0.3343	2.5097	1.1996	0.9495–1.4497	0.6193	0.3510	2.5434
Intra-operator 6	0.7510	0.6609–0.8411	0.2230	0.3040	1.2313	0.8404	0.6420–1.0388	0.4913	0.2801	2.5899
Intra-operator 7	0.7669	0.6283–0.7327	0.3431	0.2929	1.9652	0.8702	0.6694–1.0710	0.4971	0.3634	2.3483

*CI* confidence interval, *Std* standard deviation, *Min* minimum, *Max* maximum.

### Accuracy

#### Euclidean distances

The Euclidean distance (ED) between MLs and CALs was evaluated as the first measure of accuracy (Table 2). The average ED over all 26 landmarks was 1.40 mm for the unaltered sample (n = 30) and 1.76 mm for the operated sample (n = 20). The most considerable differences between MLs and CALs were found for the antegonial notches, the center of the mental foramina and the superior pole of the condyle. The operated sample showed higher mean ED when compared to the unaltered sample with EDs mainly increasing in the operated region of the mandible. The principal axes of variation between ML and CAL locations were calculated for each landmark and are shown in Supplementary Materials 9 and 10.

**Table 2:** Descriptive statistics for the Euclidean distance between the 26 MLs and CALs (mm) in the unaltered and operated sample.

	Unaltered sample (n = 30)				Operated sample (n = 20)			
	Mean	Std	Min	Max	Mean	Std	Min	Max
Right—condylar superior pole	1.85	1.02	0.14	5.20	1.81	1.12	0.13	5.32
Right—condylar medial pole	0.76	0.40	0.15	2.49	0.93	0.62	0.19	4.27
Right—condylar lateral pole	0.81	0.45	0.09	2.74	0.93	0.60	0.22	3.07
Right—condylar most posterior point	1.66	0.94	0.06	4.83	1.81	1.15	0.17	6.52
Right—condylar fovea pterygoidea center point	0.82	0.42	0.08	2.41	0.82	0.52	0.12	3.41
Right—lowest point of the incisura	1.32	1.08	0.07	5.06	0.96	0.78	0.06	3.52
Right—most superior point of the proc. coronoideus	0.67	0.41	0.10	2.18	0.86	0.70	0.08	3.58
Right—most superior point of the lingula (spix)	1.23	0.68	0.09	3.87	2.31	1.33	0.43	6.45
Right—gonion	1.46	1.17	0.08	7.07	1.64	1.21	0.20	6.51
Right—deepest point of the antegonial notch	2.19	1.56	0.15	7.99	3.18	2.23	0.31	13.07
Right—center of foramen mentale	2.13	0.93	0.23	5.78	3.24	1.96	0.61	10.50
Right—center of Tuberculum mentale	1.68	1.25	0.12	7.91	2.11	1.63	0.15	11.94
Center—protuberans mentale	1.02	0.65	0.08	3.82	1.27	0.85	0.18	4.99
Center—center point of spina mentalis	1.34	0.86	0.14	4.99	1.58	1.13	0.09	6.21
Left—center of tuberculum mentale	1.32	0.88	0.18	4.98	1.74	1.29	0.14	12.30
Left—center foramen mentale	2.41	1.03	0.56	6.24	2.47	1.22	0.64	6.80
Left—deepest point of the antegonial notch	2.11	1.51	0.13	7.97	4.39	2.93	0.16	15.49
Left—gonion	1.69	1.06	0.22	4.97	2.32	1.40	0.07	6.04
Left—most superior point of the lingula (spix)	1.22	0.70	0.23	3.88	3.32	1.82	0.39	9.34
Left—most superior point of the proc. coronoideus	0.72	0.76	0.07	7.20	0.76	0.68	0.11	3.73
Left—lowest point of the incisura	1.42	1.24	0.12	7.92	1.18	0.96	0.13	4.50
Left—condylar fovea pterygoidea center point	1.07	0.64	0.07	3.95	0.95	0.56	0.19	4.16
Left—condylar most posterior point	1.49	0.77	0.20	4.09	1.44	0.87	0.10	4.79
Left—condylar lateral pole	0.89	0.58	0.16	3.28	0.92	0.55	0.13	2.55
Left—condylar medial pole	0.91	0.57	0.11	3.22	0.79	0.40	0.14	2.14
Left—condylar superior pole	2.12	1.35	0.13	7.56	2.01	1.13	0.16	5.14
Averaged (n = 26)	1.40	0.88	0.14	5.06	1.76	1.14	0.20	6.40

*Std* standard deviation, *Min* minimum, *Max* maximum.

#### Centroid size comparison

**Figure 4:**
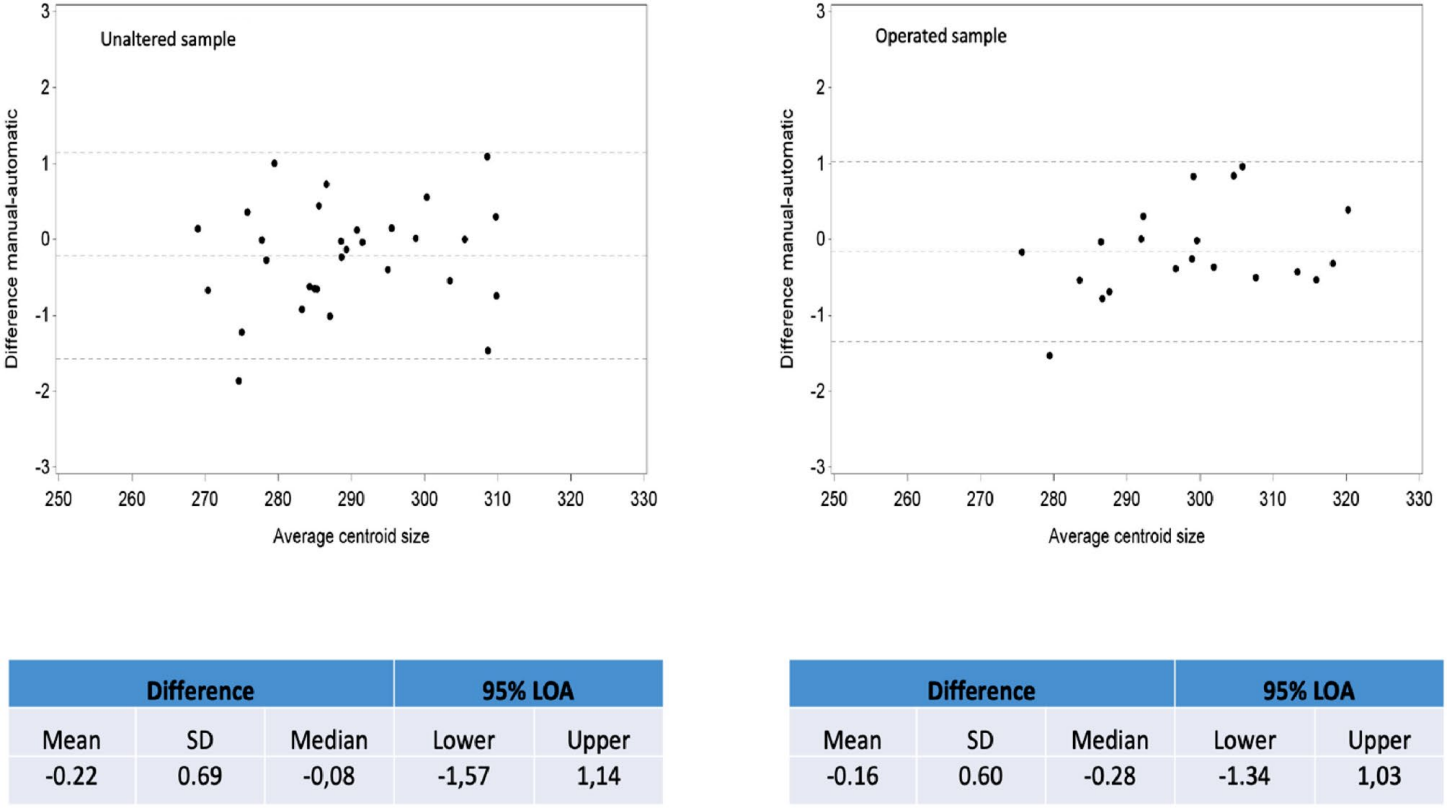
Bland–Altman plots evaluating accuracy of automatic phenotyping by assessing agreement between centroid sizes averaged over all observers resulting from landmark configurations of both methods. Left: unaltered sample. Right: operated sample.

Bland–Altman plots (Fig. 4) show a mean difference in centroid sizes of 0.22 mm for the unaltered sample (n = 30, 30 mandibles, averaged centroids over observers) and 0.16 mm for the operated sample (n = 20, 20 mandibles, averaged centroids over observers). Table 3 shows the variance components for centroid size of the mandible (random), observer (random) and method (fixed). SAS software syntax for this analysis can be found in Supplementary Material 11. These components were used to calculate the given ICC’s, SEMs and RC’s in Table 3. Excellent ICC’s of > 0.99 showed an insignificant role for the method of phenotyping (automatic vs classic). The difference in ICC between unaltered and operated mandibles was non-significant (p = 0.5075). Reproducibility coefficients were very low, e.g. < 2 mm which is < 1% of the mean centroid size of the mandibles in these two samples.

**Table 3:** Variance components from a linear mixed model on centroid sizes from automatic and classic phenotyping. Resulting variance components of method (fixed effect), jaw (random effect) and operator (random effect) were used to calculate ICC, SEM and RC.

	Unaltered sample	Operated sample	Comparison
**Variance components**			
Jaw	144.28	168.35	
Observer	0.8278	0.6861	
Method	0.284	0.4401	
**Statistics**			
SEM *(95% CI)*	0.533 (*0.498–0.573*)	0.663 (*0.61–0.727*)	p = 0.0004
RC *(95% CI)*	1.476 (*1.379–1.588*)	1.838 (*1.691–2.013*)	
ICC *(95% CI)*	0.998 (*0.997–0.999*)	0.997 (*0.995–0.999*)	p = 0.5075

*ICC* intra-class correlation, *SEM* standard error of measurement, *RC* reproducibility, *CI* 95% confidence interval. For the ICC, the CI is based on the Fishers transformation of the ICC. P-values are given for the comparison of the SEM and the ICC (both based on a *Z* test).

#### Shape similarity

Table 4 shows the resulting ICC for multivariate data applied on the set of 325 interlandmark distances for each observer as well as the averaged ICC over those observers. The averaged ICC for the unaltered mandibles was 0.949 with a slightly lower 0.915 for the operated mandibles. The ICC’s for all observers were > 0.90 in the unaltered as well as in the altered sample, indicating high similarity between shapes quantified by classic or automatic phenotyping.

**Table 4:** ICC for the multivariate dataset of 325 interlandmark distances.

Observer	Unaltered sample	Operated sample
	ICC (95% CI)	ICC (95% CI)
1	0.946 (0.923–0.955)	0.905 (0.861–0.931)
2	0.955 (0.938–0.962)	0.923 (0.887–0.943)
3	0.958 (0.941–0.964)	0.923 (0.887–0.943)
4	0.935 (0.913–0.945)	0.907 (0.867–0.934)
5	0.945 (0.921–0.953)	0.909 (0.857–0.938)
6	0.957 (0.940–0.965)	0.93 (0.896–0.948)
7	0.95 (0.928–0.958)	0.908 (0.861–0.934)
Mean	0.949 (0.929–0.957)	0.915 (0.874–0.939)

## Discussion

CMF phenotyping remains an essential tool in fundamental sciences, patient diagnosis and patient follow-up. The classic anthropometric and cephalometric approach on capturing CMF shape both have their limitations: they are time-consuming, prone for observer error and only capture a fraction of the available shape data. Automatic 3D dense CMF phenotyping using the Meshmonk toolbox overcomes these problems. In this study, we validate the use of this technique on meshes of human mandibles derived from CBCT.

This study assessed the RM reliability of automatic 3D phenotyping trying to answer the question: does a specific quasi-landmark always end up on the same position of the mandibular surface when the process is repeated? Although this is to be expected by the deterministic algorithms used, the initialisation phase of the mapping by identifying the five initialisation landmarks could introduce some variation in the mapping. The results showed very low RMS values, especially in comparison to the manual method (> ~ 100 decrease), rendering the variation of the mapping clinically insignificant.

Automatic phenotyping also showed good accuracy when compared to the classic approach, which still serves as the golden standard in practice. A mean ED of 1.40 mm and 1.76 mm was found between MLs and CALs which is in line with results from other studies on soft tissue targets^11,14,23–25^. The assessment of shape similarity between classic and automatic landmark configurations also showed promising results through high ICC’s (> 0.90) for centroid size and interlandmark distances.

This study was limited by two main factors. First of all, the absence of a robust golden standard for identifying anatomical landmarks makes it hard to illustrate the accuracy of automatic phenotyping. The results of the accuracy assessment in this study should therefore be interpreted with care as they only give insights in how automatic phenotyping compares to classic phenotyping. Secondly, we opted for 2 separate samples to assess how the automatic phenotyping performed on a more complex shape. This however resulted in smaller sample sizes of both groups (n = 30 and n = 20).

Despite of these limitations, high RM reliability and good accuracy in comparison to the current clinical standard, validate the use of automatic 3D dense CMF phenotyping on the mandible. This opens the doors for further adaptation of this approach in science and clinical practice. Patient diagnosis and follow-up could be facing a leap forward when this approach replaces anthropometric and cephalometric assessment. First of all, it allows an update on the epidemiological studies to determine what a normal shape is^16^. This data can then be used for enhanced patient diagnosis in which the CMF soft tissue and skeletal shape of a specific pathological patient are compared to the norm. Only this time, the assessment is not based on a fraction of the available shape information, but the complete shape. Patient follow-up will also be benefit from this new approach by establishing anatomical correspondence on 3D representations of the same or different patients^26^. This makes it possible to assess surgical outcomes in CMF surgery or CMF bone remodelling. Nowadays, when comparing two 3D representations of a patient, closest point analysis is mostly used. This approach identifies the closest points between two surfaces and interprets the distance between the points as the shape difference. It is, however, not a given fact that the closest point is the anatomical correspondent point. 3D CMF phenotyping provides a tool to identify anatomical correspondence on 3D models and overcome this issue.

**Figure 5:**
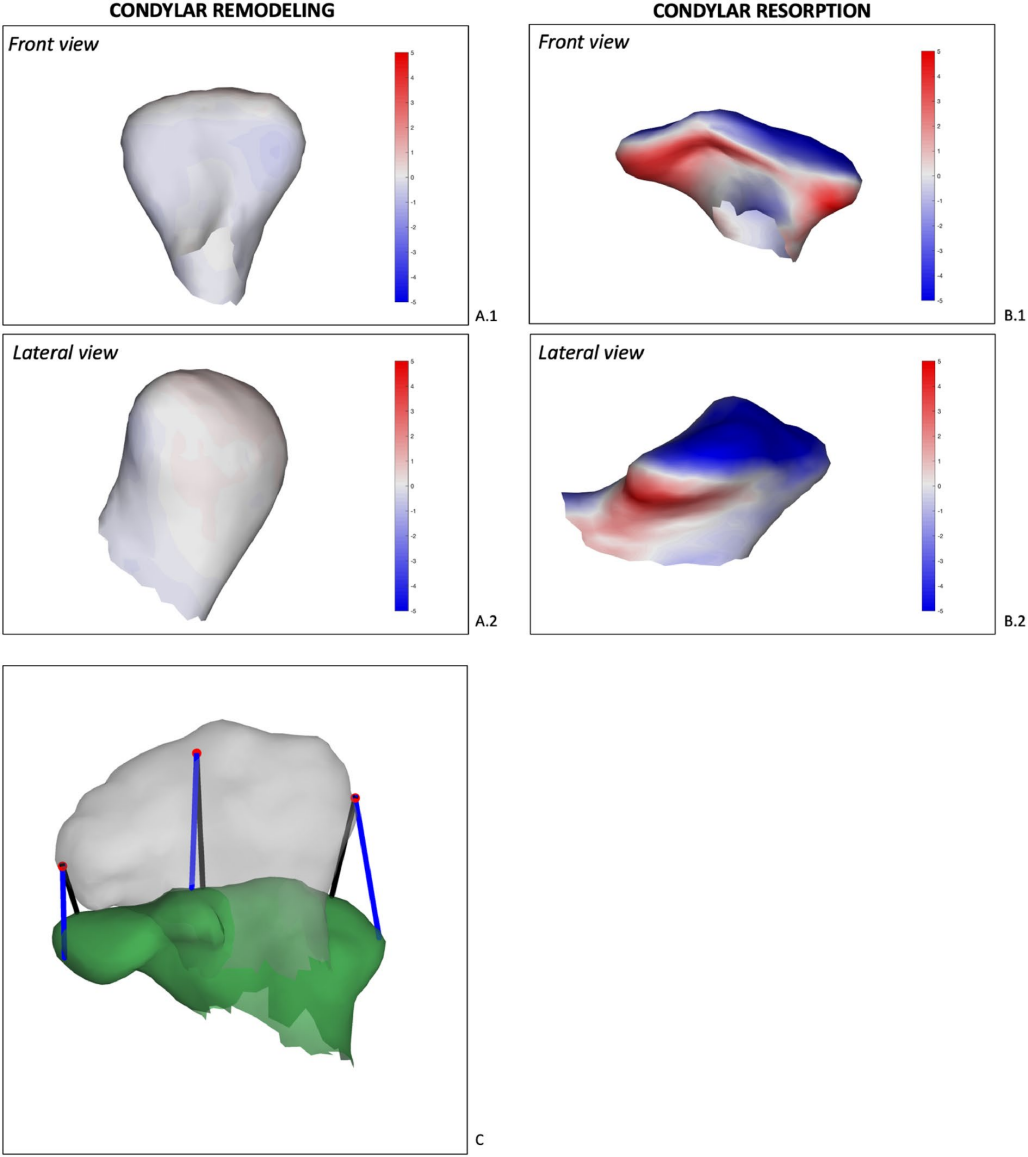
Analysis of condylar remodeling using anatomical correspondence. Heat maps are displayed on the 6-month postoperative condyles of two different patients. Blue surfaces mark bone resorption and red surfaces bone apposition with a scale in mm. Left we see a front (**A.1**) and lateral (**A.2**) view of a normal condylar remodeling case. Right (**B.1** and **B.2**) we see a case of condylar resorption with evident vertical bone loss marked by the blue surface on the superior aspect of the condyle. Panel C illustrates the difference between closest point analysis (black) and correspondent point analysis (blue) between the preoperative and postoperative condyle of patient B for three landmarks (red dots).

This is showcased in Fig. 5 where we analyse remodelling of the mandibular condyle after orthognathic surgery. After corrective surgery of the mandible, physiological remodelling of the condyle can be expected. Sometimes, this process exceeds its physiological capacity, and a pathological volume loss of the condyles is observed. This is accompanied by the lower jaw that falls back, reoccurrence of malocclusion and sometimes pain in the temporomandibular joint. 3D CMF phenotyping allows for the identification of anatomical correspondent points and allows subsequent correct quantification of how the condyle remodelled over time.

## Conclusions

This study validated automatic 3D dense CMF phenotyping of the human mandible using the Meshmonk toolbox. Excellent repeated-measures reliability and good accuracy were achieved. When combined with soft tissue phenotyping, this approach introduces an essential improvement in quantifying CMF shape. This new application of 3D dense automatic phenotyping will propel patient diagnosis and follow-up forward when implemented in diagnostic and virtual surgical planning tools.

## Supplementary Information

Supplementary Information.

## Data Availability

The Meshmonk toolbox is implemented in C++ through MATLAB and the code and tutorials are available at https://github.com/TheWebMonks/meshmonk.
