# Supplementary material for: Automatic 3D dense phenotyping provides reliable and accurate shape quantification of the human mandible
Source: Sci Rep. 2021 Apr 20;11:8532. doi: 10.1038/s41598-021-88095-w (PMC8058070; doi:10.1038/s41598-021-88095-w)
Supplement: Supplementary file 1 — Supplementary Information. [file 41598_2021_88095_MOESM1_ESM.docx]

**SUPPLEMENTARY INFORMATION**

**TITLE**

Automatic 3D dense phenotyping provides reliable and accurate shape quantification of the human mandible

**AUTHORS**

Verhelst PJ^a,b^, Matthews H^c,d,e^, Verstraete L^a,b^, Van der Cruyssen F^a,b^, Mulier D^a,b^, Croonenborghs TM^a,b^, Da Costa O^a,b^, Smeets M^a,b^, Fieuws S^f^, Shaheen E^a,b^, Jacobs R^a,b,g^, Claes P^c,d,e,h^, Politis C^a,b^, Peeters H^c,i^.

a OMFS IMPATH Research Group, Department of Imaging and Pathology, Faculty of Medicine, KU Leuven, Leuven, Belgium

b Department of Oral and Maxillofacial Surgery, University Hospitals Leuven, Leuven, Belgium

c Department of Human Genetics, KU Leuven, Leuven, Belgium

d Medical Imaging Research Center, University Hospitals Leuven, Leuven, Belgium

e Facial Sciences Research Group, Murdoch Children’s Research institute

f Leuven Biostatistics and Statistical Bioinformatics Centre, KU Leuven, Leuven, Belgium

g Department of Dental Medicine, Karolinska Institutet, Stockholm, Sweden

h Department of Electrical Engineering, ESAT/PSI, KU Leuven, Leuven, Belgium

I Department of Human Genetics, University Hospitals Leuven, Leuven, Belgium

**S1: Intra- (O1-7) and inter-operator (IO) reliability of the manual landmarking based on the RMS distance (mm) to the centroid of the three indications for intra-operator numbers and 7 indications for the inter-operator numbers. These are the numbers for the unaltered shape sample (n=30).**

| **Landmarks** | **O1** | **O2** | **O3** | **O4** | **O5** | **O6** | **O7** | **IO** |
| --- | --- | --- | --- | --- | --- | --- | --- | --- |
| 1 Right – Condylar superior pole | 1.05 | 1.33 | 0.94 | 1.70 | 1.25 | 0.65 | 0.85 | 1.47 |
| 2 Right – Condylar medial pole | 0.82 | 1.05 | 0.55 | 0.78 | 0.64 | 0.61 | 0.53 | 0.75 |
| 3 Right – Condylar lateral pole | 0.72 | 1.08 | 0.72 | 0.94 | 1.00 | 0.49 | 0.72 | 1.03 |
| 4 Right – Condylar most posterior point | 1.77 | 1.46 | 1.80 | 1.48 | 2.43 | 1.13 | 1.41 | 2.12 |
| 5 Right – Condylar fovea pterygoidea center point | 1.15 | 0.61 | 1.00 | 0.95 | 1.07 | 0.60 | 0.64 | 1.22 |
| 6 Right – Lowest point of the incisura | 0.48 | 0.67 | 0.59 | 0.66 | 0.57 | 0.46 | 0.55 | 0.65 |
| 7 Right – Most superior point of the proc. coronoideus | 0.61 | 0.42 | 0.33 | 0.56 | 0.33 | 0.30 | 0.37 | 0.49 |
| 8 Right – Most superior point of the lingula (spix) | 0.76 | 0.53 | 0.68 | 0.65 | 0.74 | 0.66 | 0.58 | 0.69 |
| 9 Right – Gonion | 1.40 | 1.81 | 1.19 | 1.78 | 1.36 | 0.85 | 1.00 | 1.82 |
| 10 Right – Deepest point of the antegonial notch | 1.22 | 1.44 | 0.92 | 1.53 | 1.38 | 0.84 | 0.84 | 1.29 |
| 11 Right – Center of foramen mentale | 0.68 | 0.77 | 0.61 | 0.68 | 0.70 | 0.55 | 0.61 | 0.55 |
| 12 Right – Center of Tuberculum mentale | 1.01 | 1.49 | 1.16 | 1.78 | 1.30 | 1.16 | 1.04 | 1.67 |
| 13 Center – Protuberans mentale | 0.87 | 1.12 | 0.80 | 1.15 | 1.02 | 0.80 | 0.95 | 0.95 |
| 14 Center – Center point of spina mentalis | 0.90 | 0.90 | 1.12 | 1.67 | 0.96 | 0.86 | 0.78 | 1.09 |
| 15 Left – Center of Tuberculum mentale | 1.26 | 1.07 | 0.88 | 1.52 | 0.95 | 0.85 | 0.72 | 1.41 |
| 16 Left – Center Foramen mentale | 0.74 | 0.53 | 0.85 | 0.90 | 0.56 | 0.59 | 0.65 | 0.67 |
| 17 Left – Deepest point of the antegonial notch | 1.38 | 1.41 | 1.04 | 2.29 | 1.54 | 0.92 | 0.95 | 1.51 |
| 18 Left – Gonion | 1.32 | 1.29 | 1.18 | 1.65 | 1.30 | 0.90 | 0.86 | 2.12 |
| 19 Left – Most superior point of the lingula (spix) | 0.99 | 0.49 | 0.88 | 0.93 | 0.91 | 0.79 | 0.48 | 0.90 |
| 20 Left – Most superior point of the proc. Coronoideus | 0.49 | 0.52 | 0.34 | 0.94 | 0.70 | 0.66 | 0.29 | 0.68 |
| 21 Left – Lowest point of the incisura | 0.55 | 0.89 | 0.51 | 0.99 | 0.83 | 0.87 | 0.40 | 0.98 |
| 22 Left – Condylar fovea pterygoidea center point | 0.79 | 0.68 | 1.22 | 0.81 | 0.78 | 0.57 | 0.66 | 1.27 |
| 23 Left – Condylar most posterior point | 1.95 | 1.75 | 1.93 | 1.13 | 2.51 | 1.23 | 1.97 | 2.03 |
| 24 Left – Condylar lateral pole | 0.91 | 1.16 | 0.61 | 0.70 | 1.13 | 0.58 | 0.68 | 1.00 |
| 25 Left – Condylar medial pole | 0.84 | 1.05 | 0.72 | 0.86 | 0.77 | 0.64 | 0.55 | 0.83 |
| 26 Left – Condylar superior pole | 1.21 | 1.53 | 1.17 | 1.39 | 1.50 | 0.95 | 0.86 | 1.45 |
| Mean | 1.00 | 1.04 | 0.91 | 1.17 | 1.09 | 0.75 | 0.77 | 1.18 |
| SD | 0.37 | 0.41 | 0.38 | 0.46 | 0.51 | 0.22 | 0.34 | 0.49 |
| Min | 0.48 | 0.42 | 0.33 | 0.56 | 0.33 | 0.30 | 0.29 | 0.49 |
| Max | 1.95 | 1.81 | 1.93 | 2.29 | 2.51 | 1.23 | 1.97 | 2.12 |

**S2: Intra- (O1-7) and inter-operator (IO) reliability of the manual landmarking based on the RMS distance (mm) to the centroid of the three indications for intra-operator numbers and 7 indications for the inter-operator numbers. These are the numbers for the operated shape sample (n=20).**

| **Landmarks** | **O1** | **O2** | **O3** | **O4** | **O5** | **O6** | **O7** | **IO** |
| --- | --- | --- | --- | --- | --- | --- | --- | --- |
| 1 Right – Condylar superior pole | 1.06 | 0.89 | 0.90 | 1.31 | 1.14 | 0.55 | 0.77 | 1.57 |
| 2 Right – Condylar medial pole | 0.64 | 1.01 | 0.68 | 0.73 | 0.75 | 0.64 | 0.51 | 0.78 |
| 3 Right – Condylar lateral pole | 0.60 | 1.27 | 0.83 | 0.93 | 0.77 | 0.51 | 0.65 | 1.08 |
| 4 Right – Condylar most posterior point | 1.55 | 1.45 | 1.69 | 1.22 | 2.29 | 0.87 | 1.94 | 2.31 |
| 5 Right – Condylar fovea pterygoidea center point | 0.68 | 0.51 | 1.11 | 0.84 | 0.89 | 0.53 | 0.64 | 1.28 |
| 6 Right – Lowest point of the incisura | 0.33 | 0.46 | 0.49 | 0.64 | 0.60 | 0.39 | 0.51 | 0.57 |
| 7 Right – Most superior point of the proc. coronoideus | 0.44 | 0.48 | 0.34 | 0.54 | 0.41 | 0.36 | 0.36 | 0.54 |
| 8 Right – Most superior point of the lingula (spix) | 0.79 | 0.74 | 1.08 | 0.71 | 0.69 | 1.03 | 0.75 | 1.03 |
| 9 Right – Gonion | 1.47 | 1.44 | 1.17 | 1.87 | 1.49 | 1.26 | 0.98 | 1.79 |
| 10 Right – Deepest point of the antegonial notch | 2.21 | 2.01 | 1.27 | 1.93 | 1.66 | 1.84 | 1.62 | 2.18 |
| 11 Right – Center of foramen mentale | 1.13 | 0.78 | 0.86 | 0.88 | 1.66 | 0.42 | 0.96 | 1.11 |
| 12 Right – Center of Tuberculum mentale | 1.04 | 1.19 | 1.14 | 1.56 | 2.27 | 1.12 | 0.65 | 2.01 |
| 13 Center – Protuberans mentale | 1.22 | 0.82 | 0.88 | 1.13 | 1.14 | 0.83 | 0.84 | 1.20 |
| 14 Center – Center point of spina mentalis | 0.86 | 1.12 | 1.41 | 1.24 | 1.25 | 1.02 | 0.66 | 1.10 |
| 15 Left – Center of Tuberculum mentale | 0.92 | 0.93 | 0.97 | 1.14 | 1.14 | 0.98 | 0.54 | 1.63 |
| 16 Left – Center Foramen mentale | 1.00 | 0.68 | 0.78 | 0.94 | 0.35 | 0.68 | 0.54 | 0.79 |
| 17 Left – Deepest point of the antegonial notch | 3.34 | 3.10 | 1.45 | 3.84 | 2.54 | 2.59 | 1.62 | 3.49 |
| 18 Left – Gonion | 1.85 | 1.52 | 1.11 | 1.51 | 1.29 | 0.88 | 1.04 | 2.58 |
| 19 Left – Most superior point of the lingula (spix) | 1.65 | 1.17 | 1.47 | 0.94 | 1.31 | 0.84 | 1.06 | 1.48 |
| 20 Left – Most superior point of the proc. Coronoideus | 0.38 | 0.56 | 0.32 | 0.46 | 0.45 | 0.28 | 0.39 | 0.48 |
| 21 Left – Lowest point of the incisura | 0.47 | 0.70 | 0.57 | 0.56 | 0.56 | 0.68 | 0.56 | 0.95 |
| 22 Left – Condylar fovea pterygoidea center point | 0.92 | 0.60 | 1.51 | 0.97 | 0.88 | 0.52 | 0.68 | 1.31 |
| 23 Left – Condylar most posterior point | 1.83 | 1.62 | 2.33 | 1.31 | 2.18 | 1.13 | 2.35 | 1.95 |
| 24 Left – Condylar lateral pole | 0.64 | 1.27 | 0.53 | 0.97 | 1.11 | 0.57 | 0.54 | 1.02 |
| 25 Left – Condylar medial pole | 0.79 | 1.21 | 0.58 | 0.76 | 0.73 | 0.58 | 0.44 | 0.86 |
| 26 Left – Condylar superior pole | 1.26 | 0.98 | 0.99 | 1.61 | 1.66 | 0.77 | 1.05 | 1.43 |
| Mean | 1.12 | 1.10 | 1.02 | 1.18 | 1.20 | 0.84 | 0.87 | 1.40 |
| SD | 0.67 | 0.57 | 0.46 | 0.67 | 0.62 | 0.49 | 0.50 | 0.70 |
| Min | 0.33 | 0.46 | 0.32 | 0.46 | 0.35 | 0.28 | 0.36 | 0.48 |
| Max | 3.34 | 3.10 | 2.33 | 3.84 | 2.54 | 2.59 | 2.35 | 3.49 |

**S3: RMS distances (mm) to the centroid for each CAL as assessed for repeated-measures reliability of automatic mapping over 3 iterations of mapping (n=3) in the unaltered (n=30) and operated (n=20) sample.**

| **Landmarks** | **Unaltered** | **Operated** |
| --- | --- | --- |
| 1 Right – Condylar superior pole | 0.0055 | 0.0031 |
| 2 Right – Condylar medial pole | 0.0024 | 0.0021 |
| 3 Right – Condylar lateral pole | 0.0025 | 0.0021 |
| 4 Right – Condylar most posterior point | 0.0067 | 0.0038 |
| 5 Right – Condylar fovea pterygoidea center point | 0.0027 | 0.0018 |
| 6 Right – Lowest point of the incisura | 0.0022 | 0.0025 |
| 7 Right – Most superior point of the proc. coronoideus | 0.0023 | 0.0017 |
| 8 Right – Most superior point of the lingula (spix) | 0.0031 | 0.0085 |
| 9 Right – Gonion | 0.0217 | 0.0159 |
| 10 Right – Deepest point of the antegonial notch | 0.0217 | 0.0173 |
| 11 Right – Center of foramen mentale | 0.0111 | 0.0122 |
| 12 Right – Center of Tuberculum mentale | 0.0084 | 0.0079 |
| 13 Center – Protuberans mentale | 0.0069 | 0.0164 |
| 14 Center – Center point of spina mentalis | 0.0040 | 0.0036 |
| 15 Left – Center of Tuberculum mentale | 0.0061 | 0.0080 |
| 16 Left – Center Foramen mentale | 0.0187 | 0.0170 |
| 17 Left – Deepest point of the antegonial notch | 0.0160 | 0.0318 |
| 18 Left – Gonion | 0.0075 | 0.0220 |
| 19 Left – Most superior point of the lingula (spix) | 0.0025 | 0.0078 |
| 20 Left – Most superior point of the proc. Coronoideus | 0.0022 | 0.0021 |
| 21 Left – Lowest point of the incisura | 0.0056 | 0.0029 |
| 22 Left – Condylar fovea pterygoidea center point | 0.0027 | 0.0021 |
| 23 Left – Condylar most posterior point | 0.0045 | 0.0021 |
| 24 Left – Condylar lateral pole | 0.0019 | 0.0016 |
| 25 Left – Condylar medial pole | 0.0021 | 0.0012 |
| 26 Left – Condylar superior pole | 0.0038 | 0.0028 |
| Mean | 0.0067 | 0.0077 |
| SD | 0.0061 | 0.0079 |
| Min | 0.0019 | 0.0012 |
| Max | 0.0217 | 0.0318 |

**S4: color scale illustrating the reliability of the automatic mapping over all 17415 quasi landmarks.**


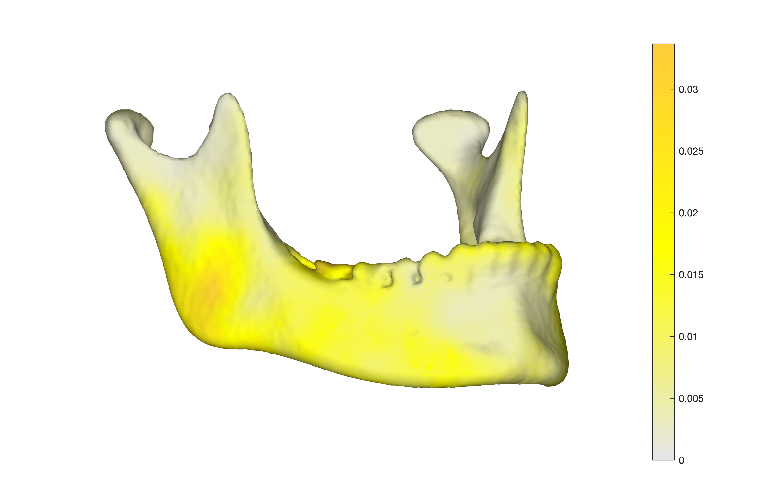

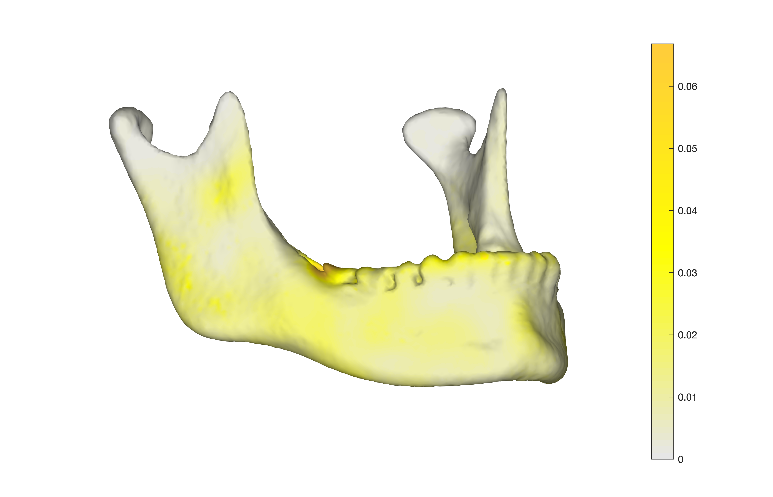

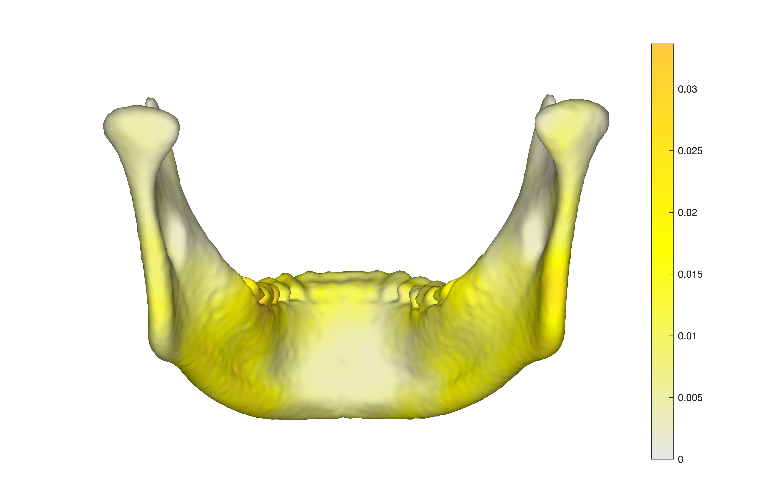

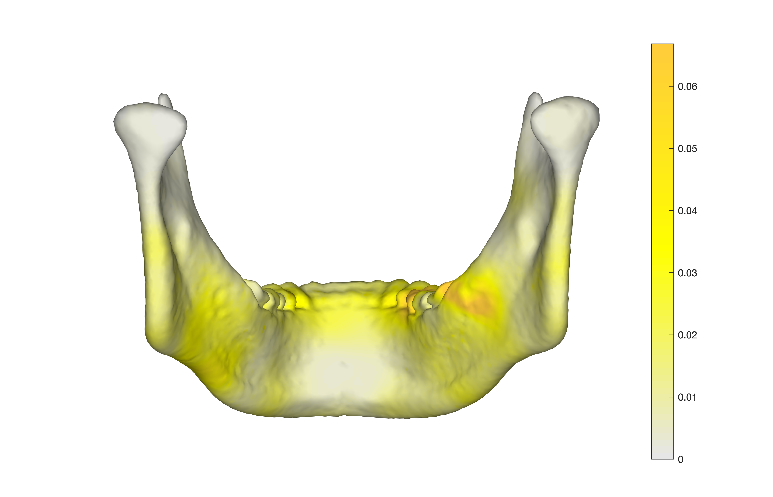

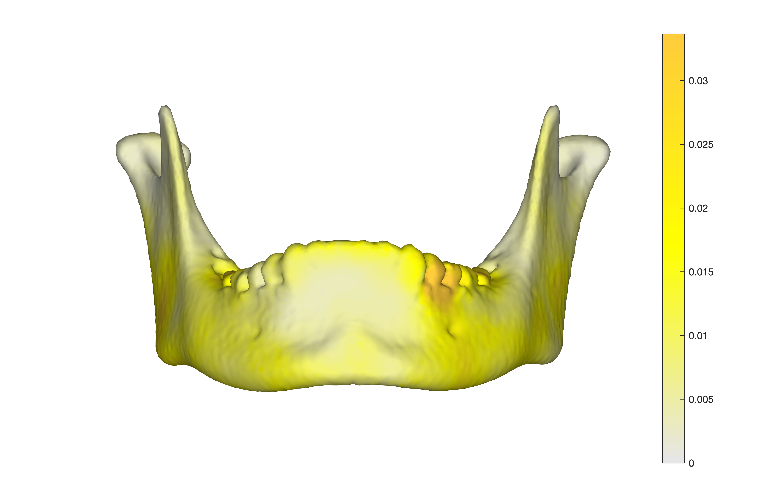

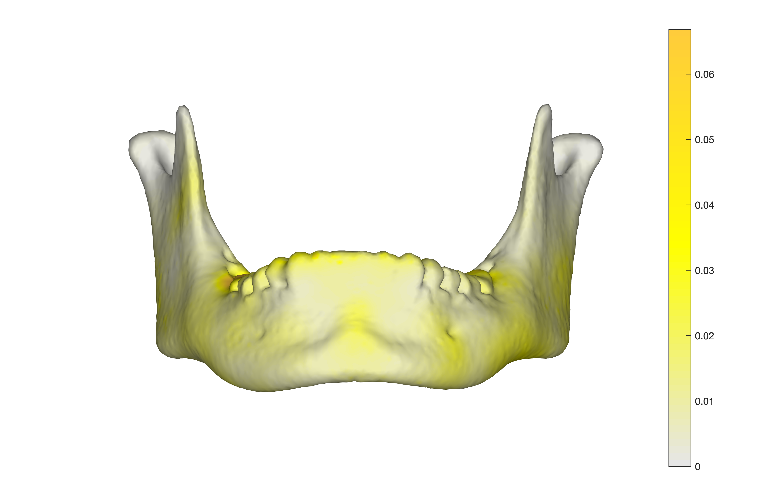
  **Unaltered shape (n=30)**  **Operated shape (n=20)**

**These figures illustrate the error of the automatic phenotyping, being the mapping of the 17415 quasi landmarks of the template mesh on a target mandible. It serves as a measure of the automatic phenotyping reliability. The scale on the right side is the RMS distance (mm) to the centroid of the three indications of each quasi-landmark (n=17415). Note that the scale of the unaltered and operated sample differs in range. More yellow regions are those regions that show the highest variation in automatic mapping results. The table below gives the mean RMS distance over all quasi-landmarks, as well as the Std, min and max value in both samples.**

|  | Unaltered sample | | | | | Operated sample | | | | |
| --- | --- | --- | --- | --- | --- | --- | --- | --- | --- | --- |
|  | Mean | 95% CI Mean | Std | Min | Max | Mean | 95% CI Mean | Std | Min | Max |
| Automated | 0.0137 | 0.0133-0.0141 | 0.0261 | 0.0016 | 0.6031 | 0.0153 | 0.0151 - 0.0155 | 0.0117 | 0.0010 | 0.0923 |

**S5: Variation of the intra-operator error in the unaltered shape sample of the 26 manually placed landmarks. The illustrations provide a visual overview of the direction in which the most variation/error of landmark identification was observed. The three largest dimensions are shown for each landmark individually with red being the largest dimension, blue the second and green the third largest dimension. The table lists the magnitude of this variation in mm.**

| **Landmark** | **Red** | **Blue** | **Green** |
| --- | --- | --- | --- |
| 1 | 1.21 | 0.63 | 0.21 |
| 2 | 0.61 | 0.53 | 0.17 |
| 3 | 0.65 | 0.61 | 0.23 |
| 4 | 1.61 | 1.04 | 0.34 |
| 5 | 0.69 | 0.65 | 0.24 |
| 6 | 0.69 | 0.17 | 0.13 |
| 7 | 0.41 | 0.22 | 0.15 |
| 8 | 0.77 | 0.34 | 0.25 |
| 9 | 1.51 | 0.46 | 0.22 |
| 10 | 1.33 | 0.41 | 0.15 |
| 11 | 0.70 | 0.43 | 0.26 |
| 12 | 1.52 | 0.53 | 0.25 |
| 13 | 0.93 | 0.52 | 0.16 |
| 14 | 1.16 | 0.57 | 0.35 |
| 15 | 1.17 | 0.55 | 0.19 |
| 16 | 0.87 | 0.53 | 0.29 |
| 17 | 1.65 | 0.42 | 0.12 |
| 18 | 1.33 | 0.42 | 0.24 |
| 19 | 0.94 | 0.34 | 0.26 |
| 20 | 1.13 | 0.39 | 0.22 |
| 21 | 1.24 | 0.40 | 0.16 |
| 22 | 0.67 | 0.58 | 0.20 |
| 23 | 1.76 | 1.05 | 0.34 |
| 24 | 0.75 | 0.55 | 0.23 |
| 25 | 0.66 | 0.57 | 0.22 |
| 26 | 1.35 | 0.63 | 0.21 |


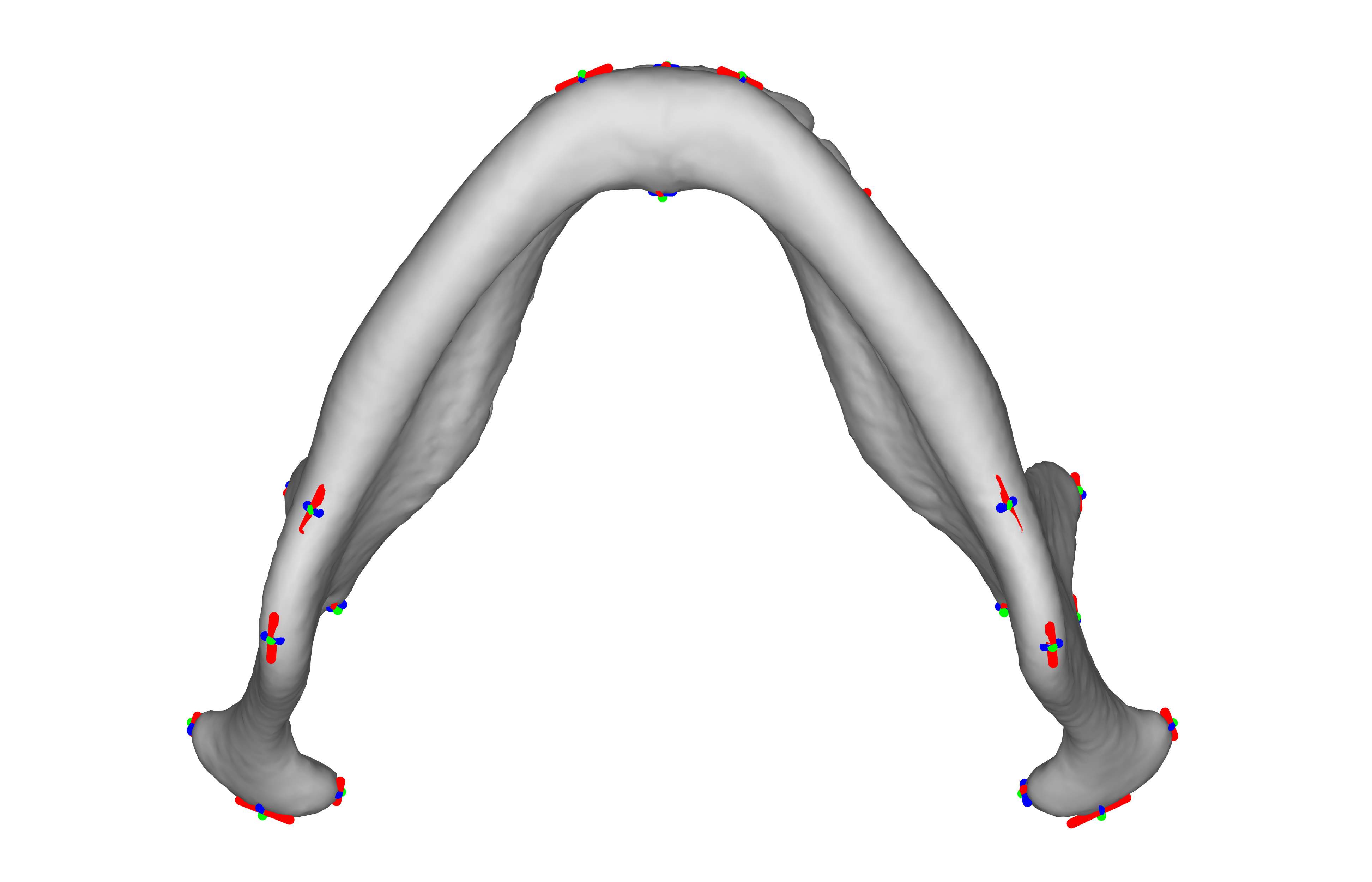

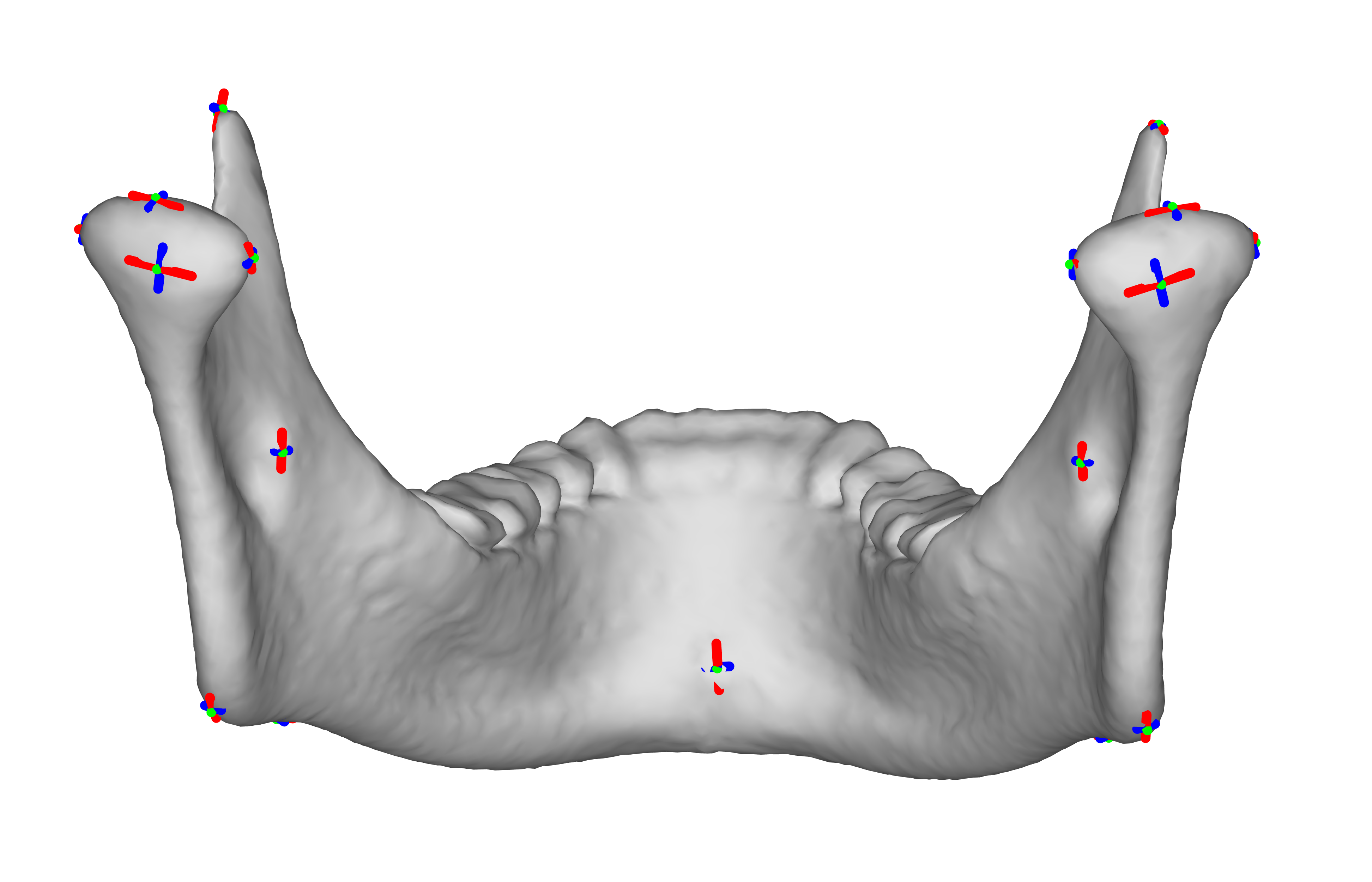

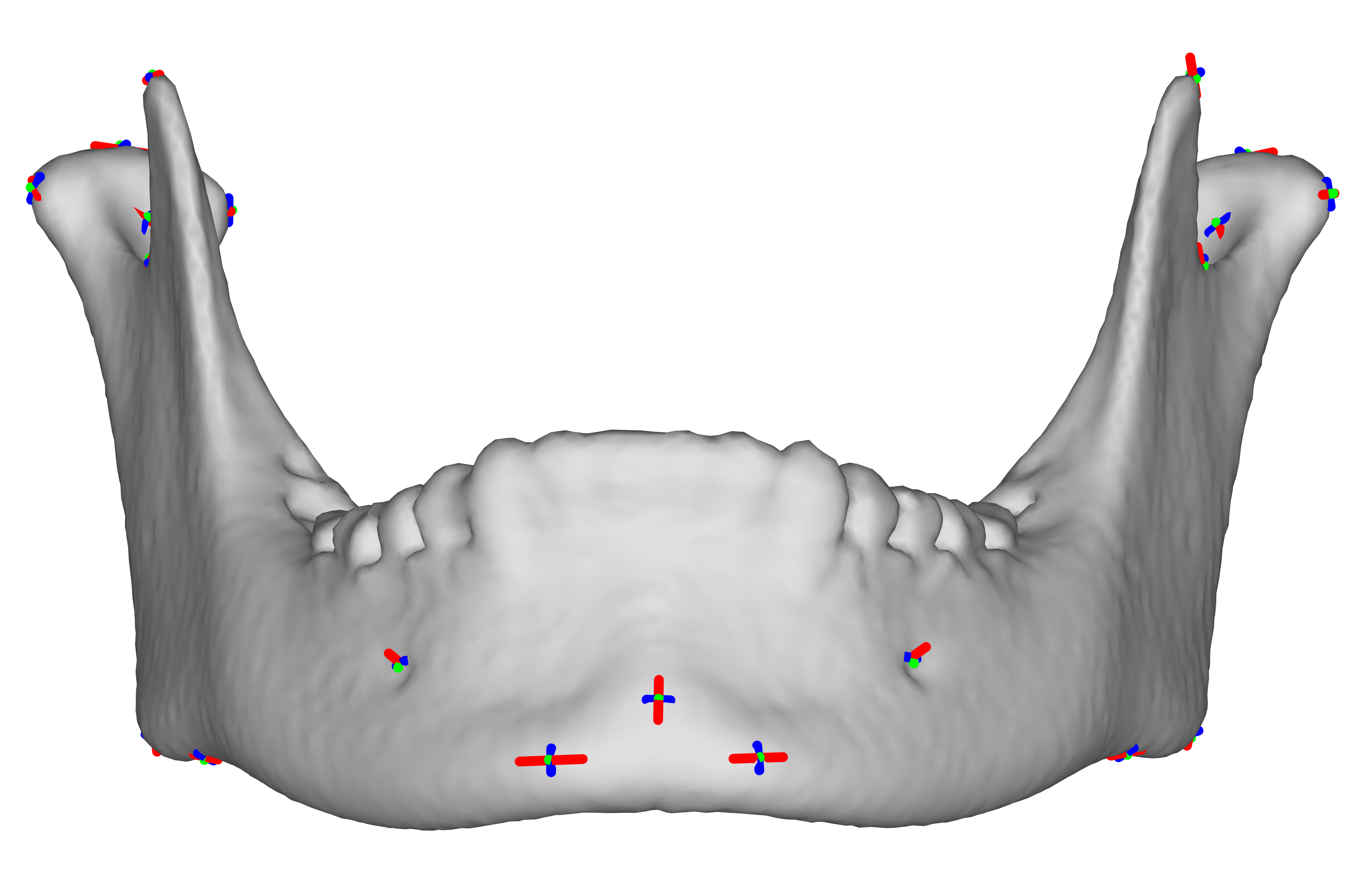


| **Landmark** | **Red** | **Blue** | **Green** |
| --- | --- | --- | --- |
| 1 | 1.24 | 0.85 | 0.18 |
| 2 | 0.55 | 0.53 | 0.14 |
| 3 | 0.80 | 0.70 | 0.20 |
| 4 | 1.91 | 1.02 | 0.30 |
| 5 | 1.07 | 0.58 | 0.25 |
| 6 | 0.72 | 0.20 | 0.10 |
| 7 | 0.44 | 0.24 | 0.11 |
| 8 | 0.66 | 0.33 | 0.19 |
| 9 | 1.82 | 0.57 | 0.23 |
| 10 | 1.32 | 0.52 | 0.11 |
| 11 | 0.51 | 0.31 | 0.22 |
| 12 | 1.78 | 0.57 | 0.22 |
| 13 | 0.89 | 0.38 | 0.16 |
| 14 | 1.08 | 0.40 | 0.29 |
| 15 | 1.40 | 0.59 | 0.17 |
| 16 | 0.65 | 0.43 | 0.22 |
| 17 | 1.66 | 0.40 | 0.11 |
| 18 | 2.13 | 0.51 | 0.24 |
| 19 | 0.90 | 0.32 | 0.21 |
| 20 | 0.78 | 0.41 | 0.21 |
| 21 | 1.03 | 0.38 | 0.20 |
| 22 | 0.97 | 0.89 | 0.29 |
| 23 | 1.83 | 1.01 | 0.28 |
| 24 | 0.85 | 0.59 | 0.19 |
| 25 | 0.69 | 0.52 | 0.17 |
| 26 | 1.22 | 0.90 | 0.18 |

**S6: Variation of the inter-operator error in the unaltered shape sample of the 26 manually placed landmarks. The illustrations provide a visual overview of the direction in which the most variation/error of landmark identification was observed. The three largest dimensions are shown for each landmark individually with red being the largest dimension, blue the second and green the third largest dimension. The table lists the magnitude of this variation in mm.**


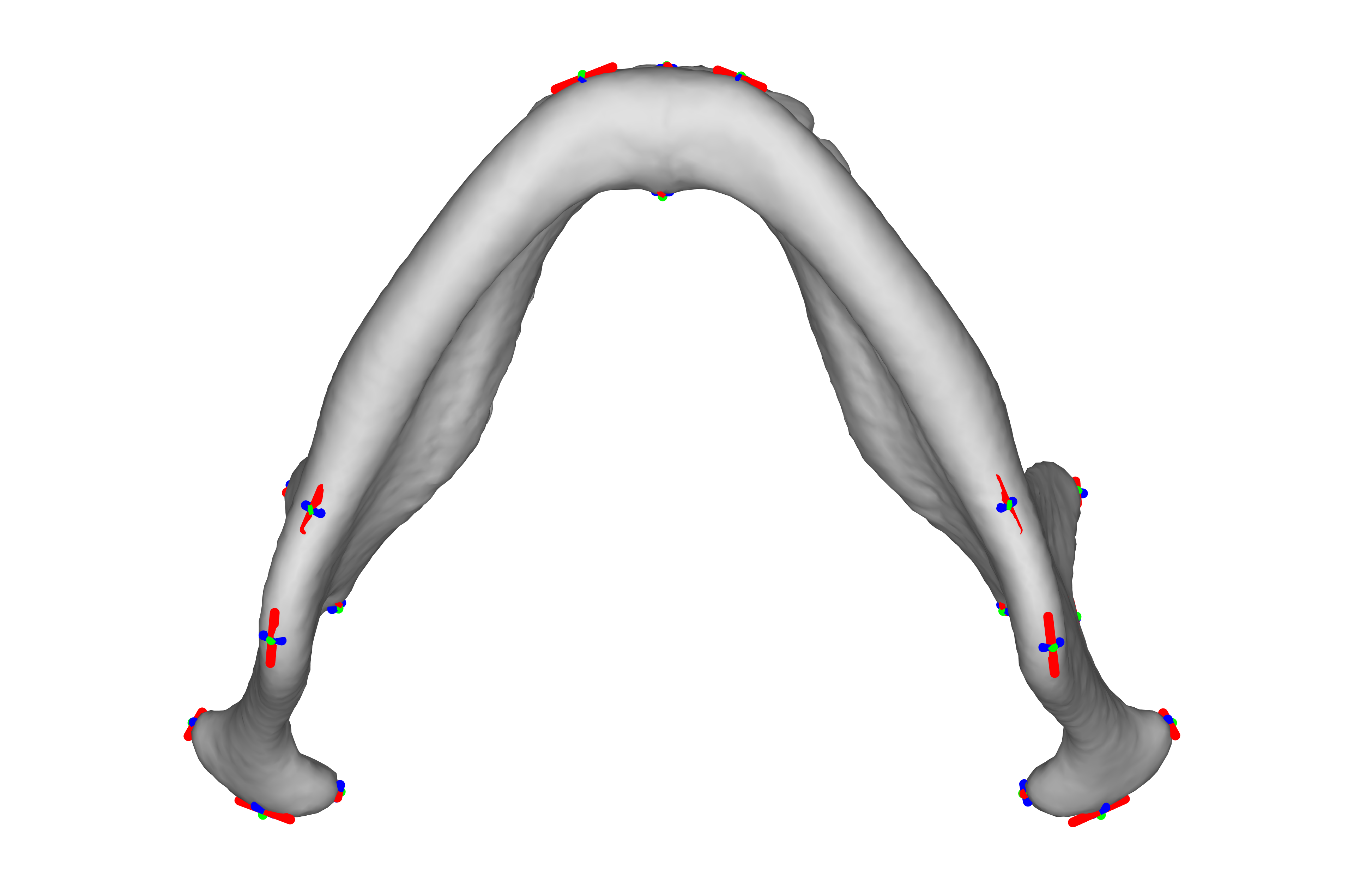

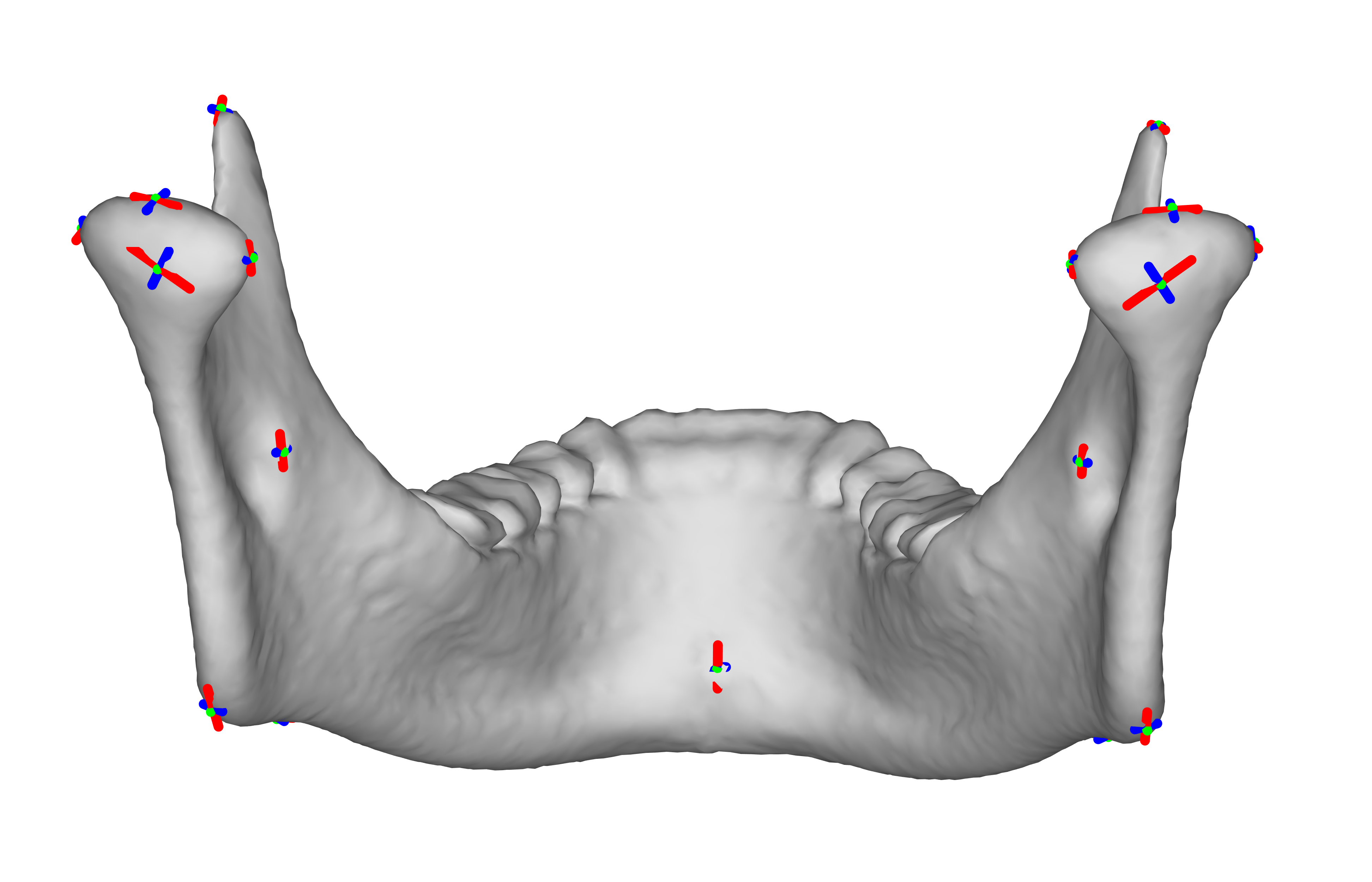

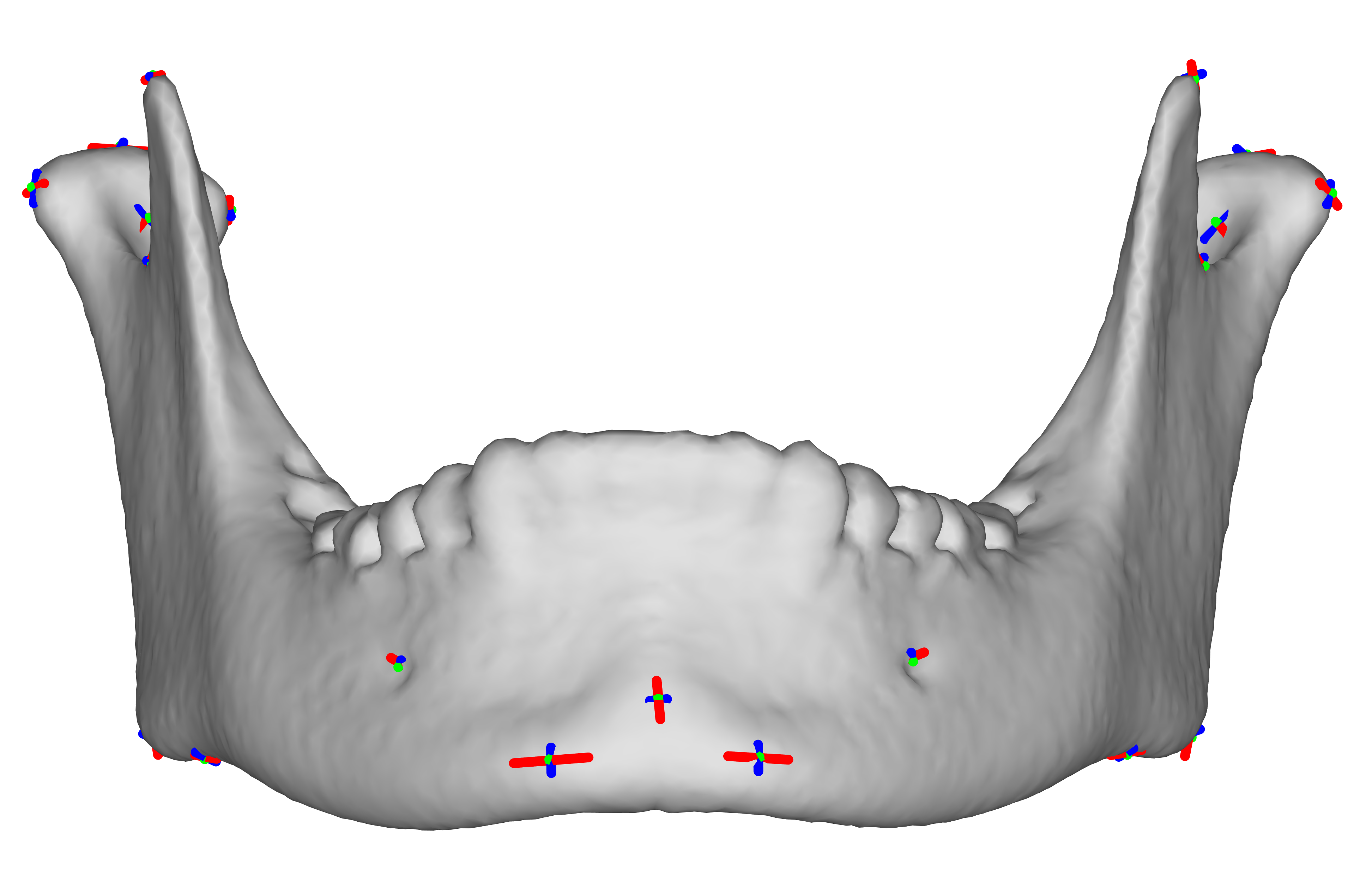


| **Landmark** | **Red** | **Blue** | **Green** |
| --- | --- | --- | --- |
| 1 | 0.99 | 0.56 | 0.19 |
| 2 | 0.63 | 0.51 | 0.15 |
| 3 | 0.66 | 0.60 | 0.30 |
| 4 | 1.49 | 1.06 | 0.36 |
| 5 | 0.64 | 0.49 | 0.22 |
| 6 | 0.54 | 0.17 | 0.15 |
| 7 | 0.39 | 0.25 | 0.21 |
| 8 | 0.99 | 0.47 | 0.26 |
| 9 | 1.48 | 0.51 | 0.22 |
| 10 | 2.26 | 0.55 | 0.19 |
| 11 | 1.35 | 0.60 | 0.46 |
| 12 | 1.55 | 0.55 | 0.27 |
| 13 | 0.84 | 0.74 | 0.23 |
| 14 | 1.03 | 0.61 | 0.41 |
| 15 | 1.06 | 0.49 | 0.22 |
| 16 | 0.85 | 0.58 | 0.33 |
| 17 | 3.79 | 0.59 | 0.37 |
| 18 | 1.42 | 0.51 | 0.27 |
| 19 | 1.48 | 0.77 | 0.42 |
| 20 | 0.39 | 0.19 | 0.18 |
| 21 | 0.67 | 0.19 | 0.15 |
| 22 | 0.72 | 0.65 | 0.23 |
| 23 | 1.82 | 1.11 | 0.38 |
| 24 | 0.72 | 0.57 | 0.26 |
| 25 | 0.60 | 0.56 | 0.20 |
| 26 | 1.25 | 0.68 | 0.27 |

**S7: Variation of the intra-operator error in the operated shape sample of the 26 manually placed landmarks. The illustrations provide a visual overview of the direction in which the most variation/error of landmark identification was observed. The three largest dimensions are shown for each landmark individually with red being the largest dimension, blue the second and green the third largest dimension. The table lists the magnitude of this variation in mm.**


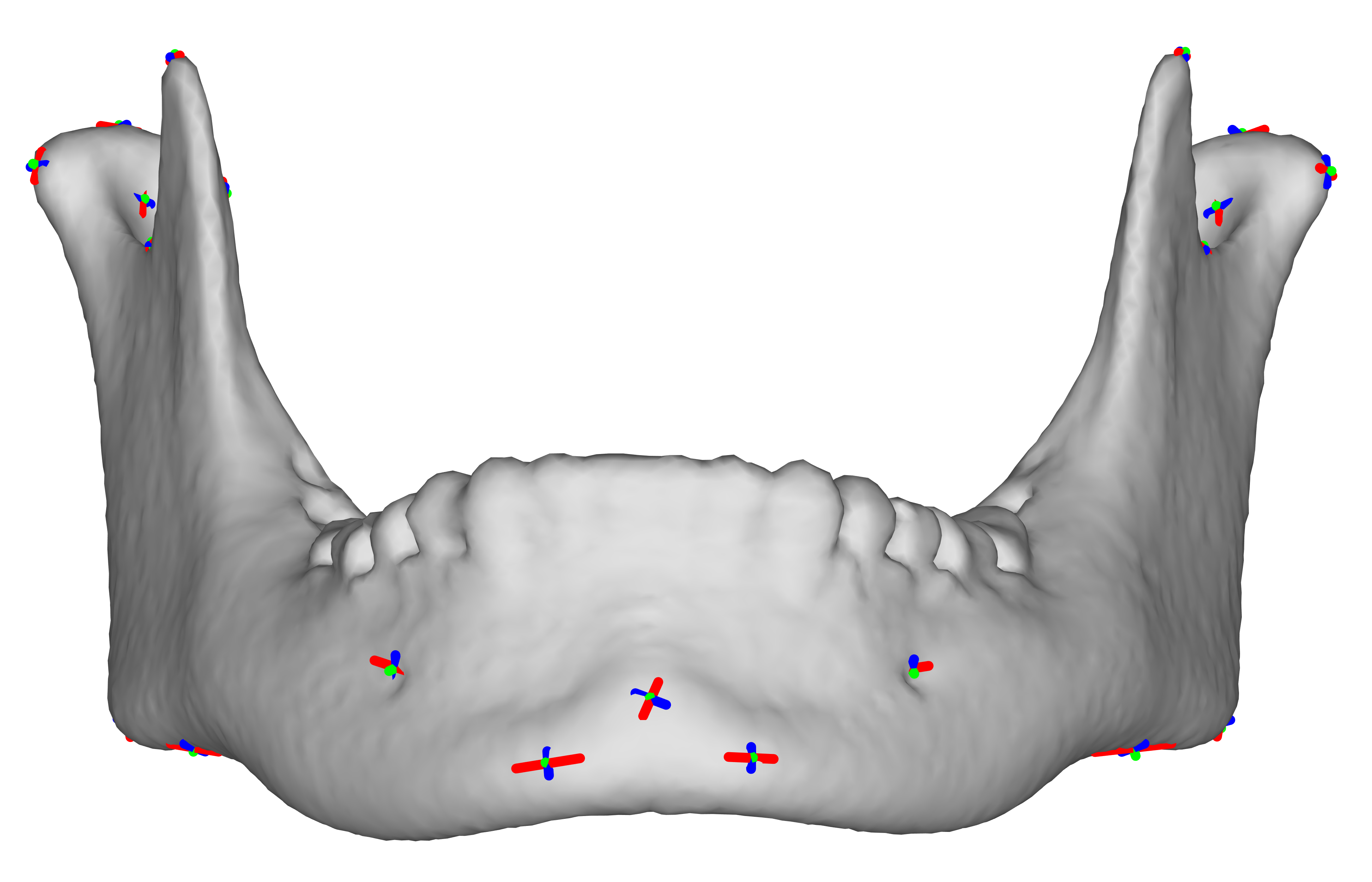

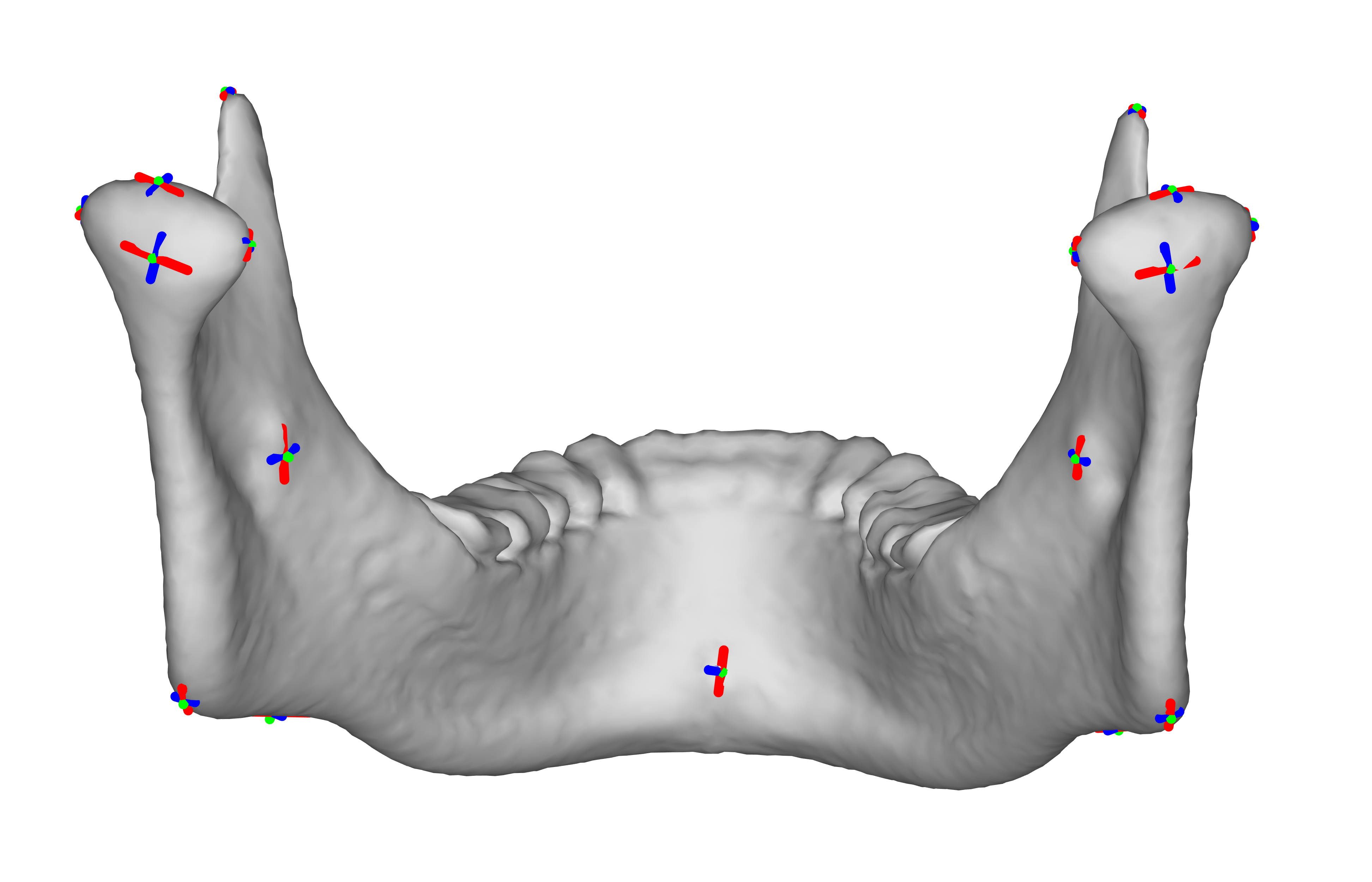

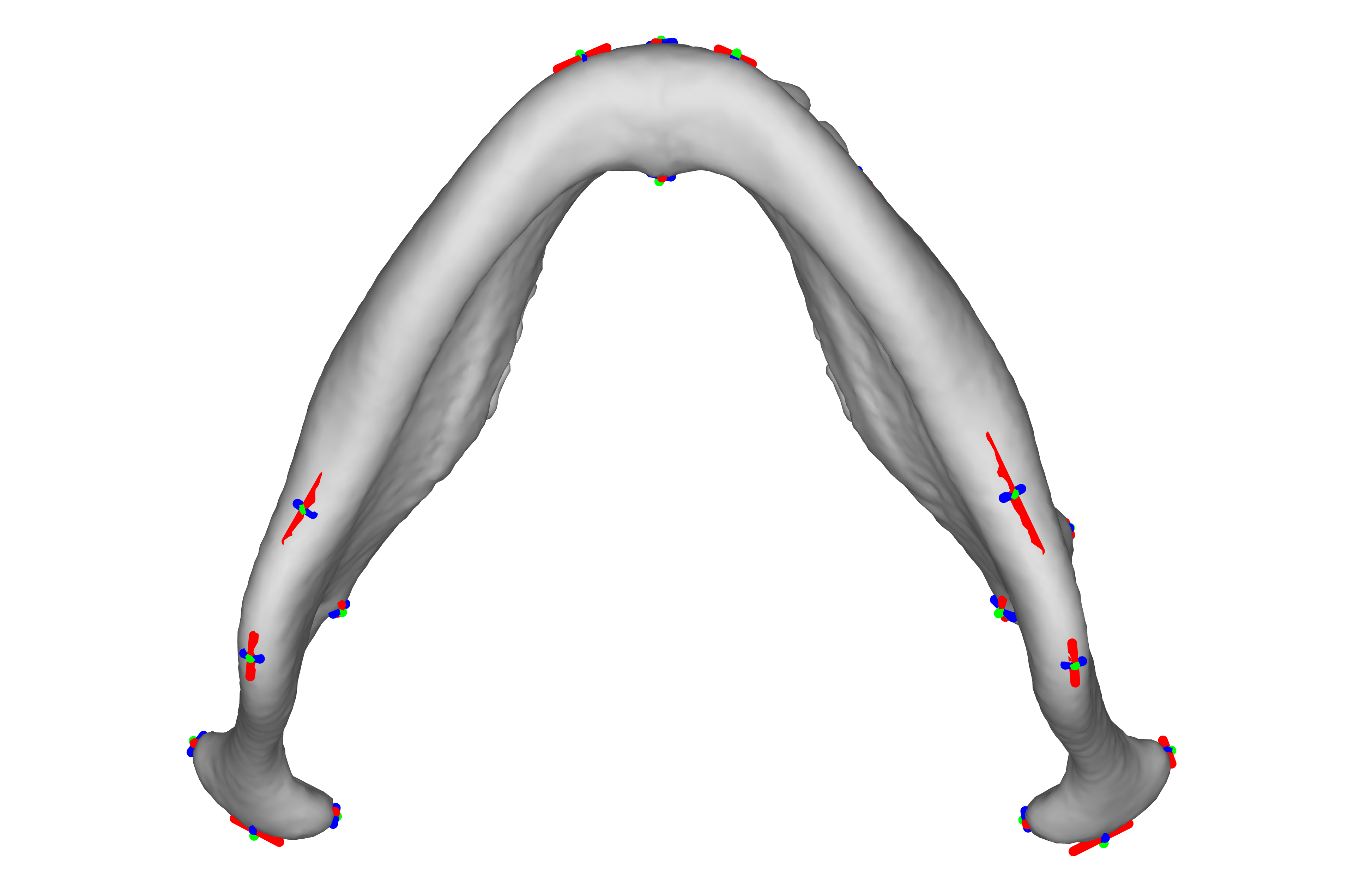


**S8: Variation of the inter-operator error in the operated shape sample of the 26 manually placed landmarks. The illustrations provide a visual overview of the direction in which the most variation/error of landmark identification was observed. The three largest dimensions are shown for each landmark individually with red being the largest dimension, blue the second and green the third largest dimension. The table lists the magnitude of this variation in mm.**


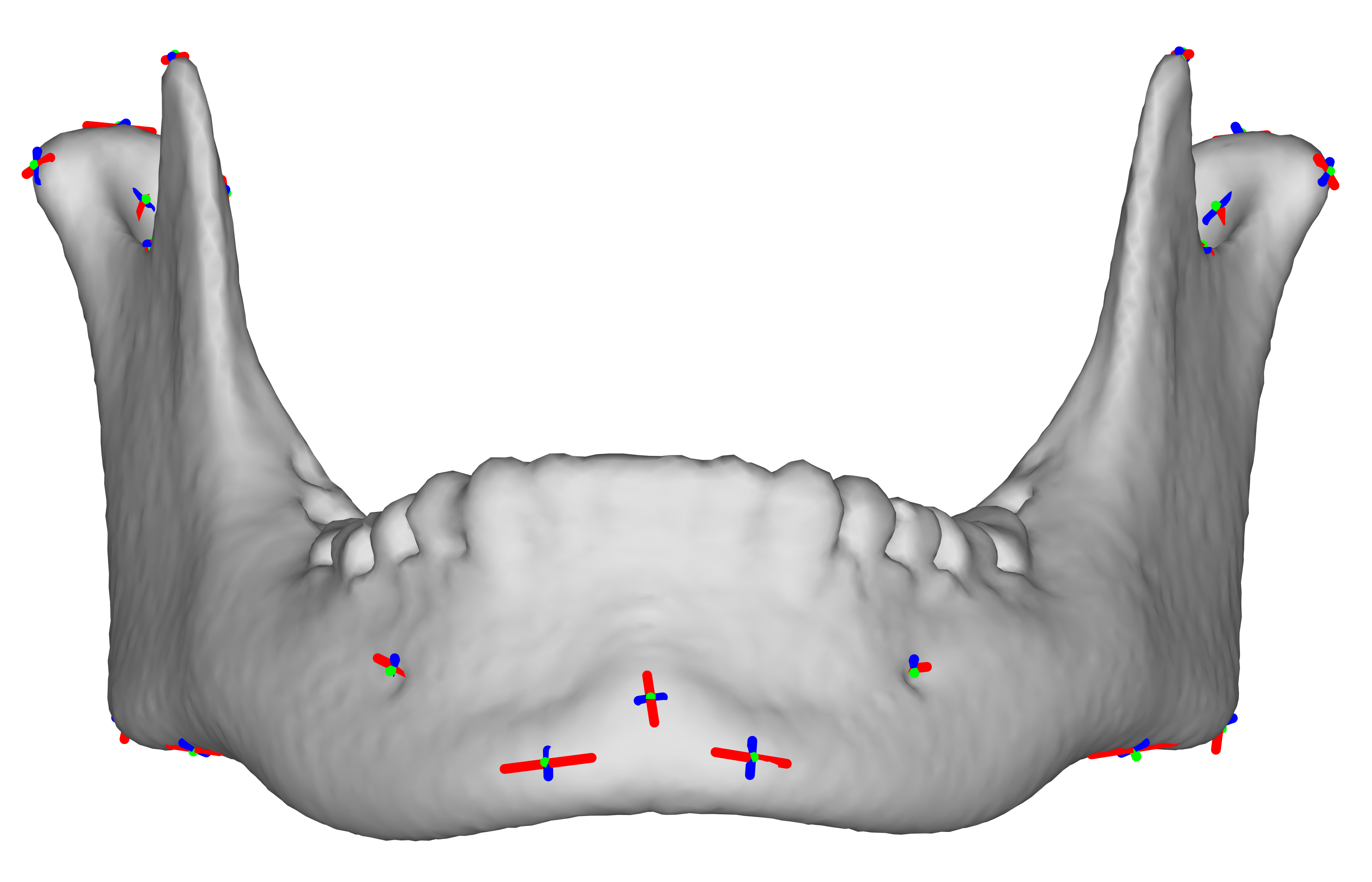


| **Landmark** | **Red** | **Blue** | **Green** |
| --- | --- | --- | --- |
| 1 | 1.53 | 0.74 | 0.14 |
| 2 | 0.63 | 0.51 | 0.13 |
| 3 | 0.88 | 0.71 | 0.21 |
| 4 | 2.13 | 1.14 | 0.38 |
| 5 | 1.13 | 0.58 | 0.26 |
| 6 | 0.52 | 0.26 | 0.12 |
| 7 | 0.47 | 0.28 | 0.17 |
| 8 | 1.05 | 0.41 | 0.23 |
| 9 | 1.81 | 0.57 | 0.19 |
| 10 | 2.41 | 0.61 | 0.18 |
| 11 | 1.29 | 0.48 | 0.38 |
| 12 | 2.13 | 0.58 | 0.30 |
| 13 | 1.10 | 0.54 | 0.25 |
| 14 | 1.03 | 0.48 | 0.37 |
| 15 | 1.74 | 0.77 | 0.27 |
| 16 | 0.78 | 0.53 | 0.32 |
| 17 | 4.05 | 0.63 | 0.42 |
| 18 | 2.58 | 0.64 | 0.30 |
| 19 | 1.55 | 0.56 | 0.31 |
| 20 | 0.34 | 0.34 | 0.12 |
| 21 | 0.96 | 0.24 | 0.15 |
| 22 | 1.03 | 0.79 | 0.27 |
| 23 | 1.76 | 1.02 | 0.31 |
| 24 | 0.83 | 0.65 | 0.20 |
| 25 | 0.70 | 0.55 | 0.21 |
| 26 | 1.16 | 0.93 | 0.25 |


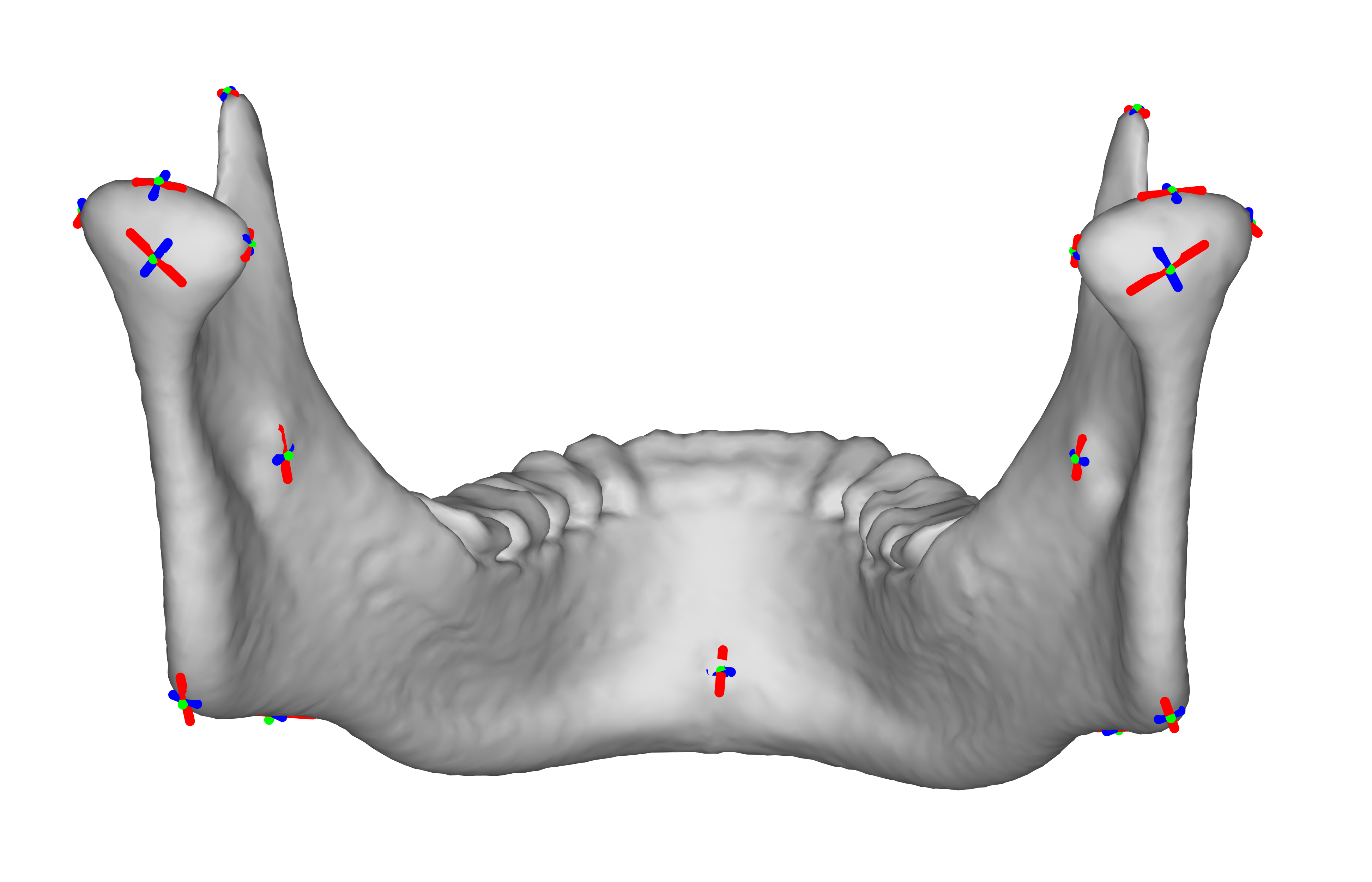

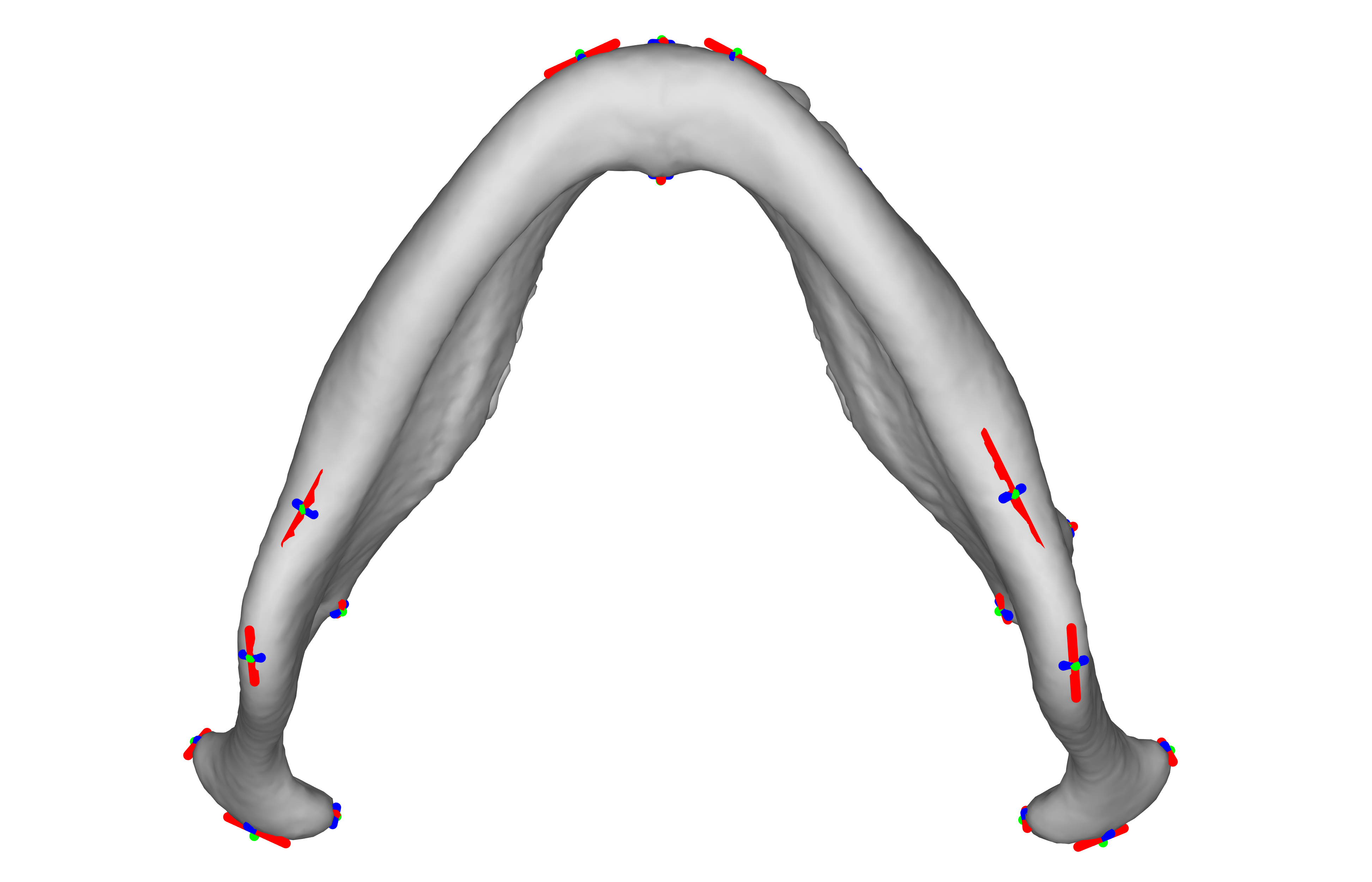


**S9: Variation in error between corresponding automatic and manually identified landmarks for the unaltered shape sample. The illustrations provide a visual overview of the direction in which the most variation/error of landmark identification was observed. The three largest dimensions are shown for each landmark individually with red being the largest dimension, blue the second and green the third largest dimension. The table lists the magnitude of this variation in mm.**


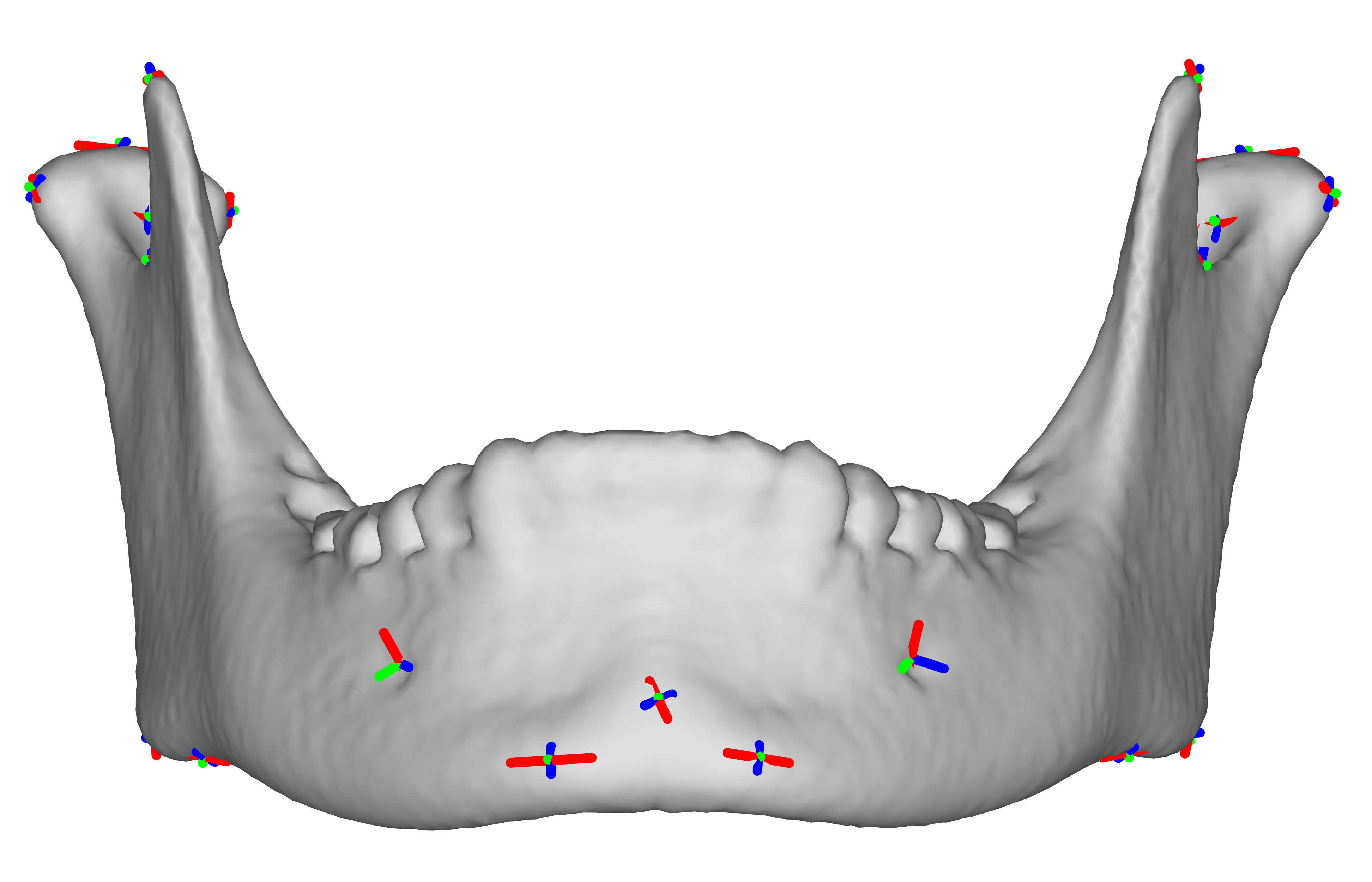


| **Landmark** | **Red** | **Blue** | **Green** |
| --- | --- | --- | --- |
| 1 | 1.94 | 0.74 | 0.37 |
| 2 | 0.61 | 0.55 | 0.25 |
| 3 | 0.67 | 0.56 | 0.32 |
| 4 | 1.64 | 0.91 | 0.35 |
| 5 | 0.67 | 0.55 | 0.29 |
| 6 | 1.64 | 0.36 | 0.25 |
| 7 | 0.55 | 0.50 | 0.25 |
| 8 | 1.18 | 0.58 | 0.50 |
| 9 | 1.80 | 0.41 | 0.32 |
| 10 | 2.62 | 0.49 | 0.30 |
| 11 | 1.58 | 1.29 | 1.13 |
| 12 | 1.98 | 0.61 | 0.32 |
| 13 | 0.98 | 0.66 | 0.27 |
| 14 | 1.33 | 0.71 | 0.50 |
| 15 | 1.45 | 0.58 | 0.26 |
| 16 | 1.67 | 1.54 | 1.31 |
| 17 | 2.54 | 0.46 | 0.26 |
| 18 | 1.90 | 0.46 | 0.37 |
| 19 | 1.17 | 0.63 | 0.43 |
| 20 | 0.89 | 0.48 | 0.26 |
| 21 | 1.70 | 0.77 | 0.27 |
| 22 | 0.98 | 0.67 | 0.39 |
| 23 | 1.41 | 0.81 | 0.39 |
| 24 | 0.77 | 0.65 | 0.34 |
| 25 | 0.84 | 0.60 | 0.31 |
| 26 | 2.38 | 0.70 | 0.39 |


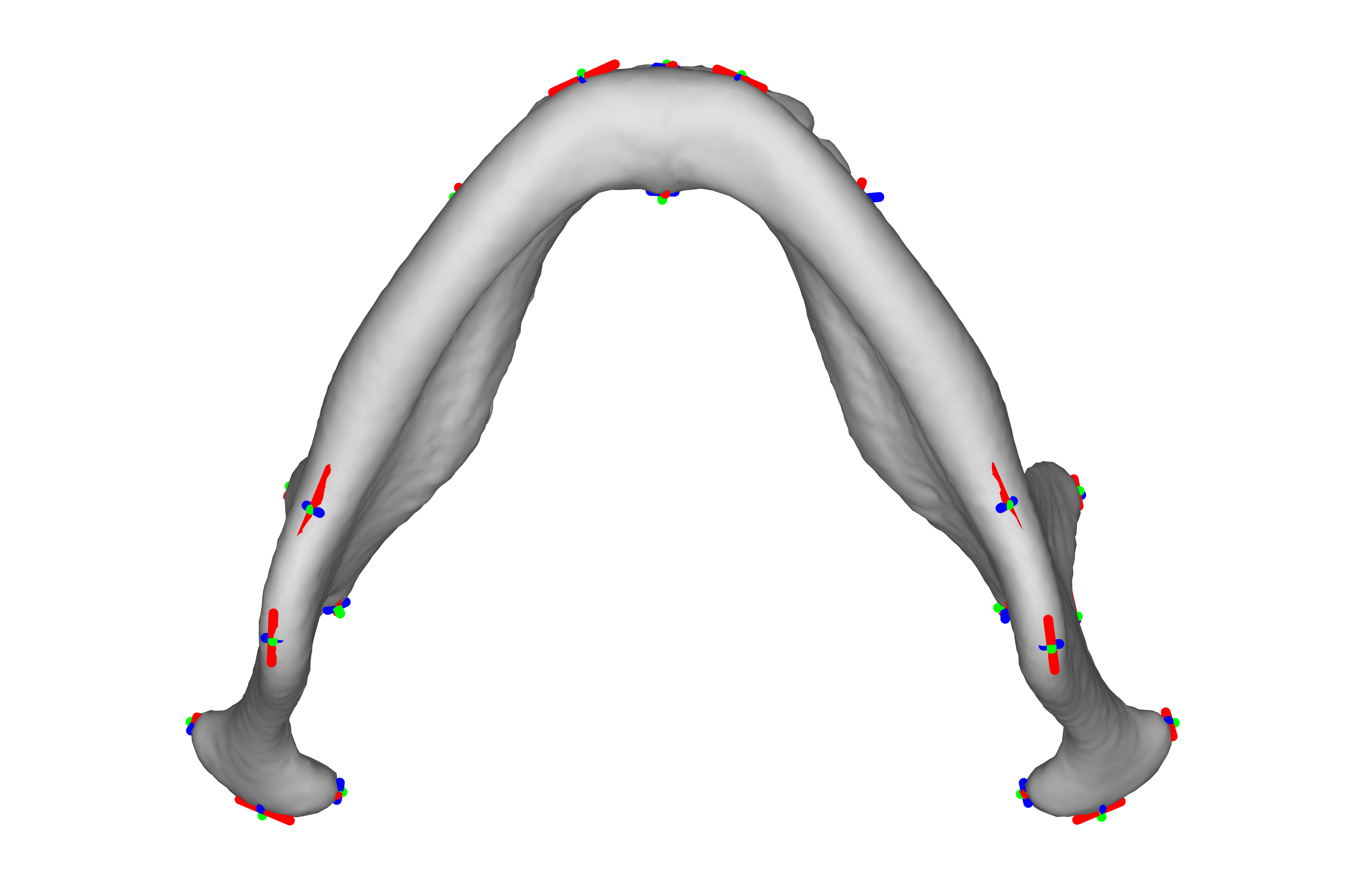

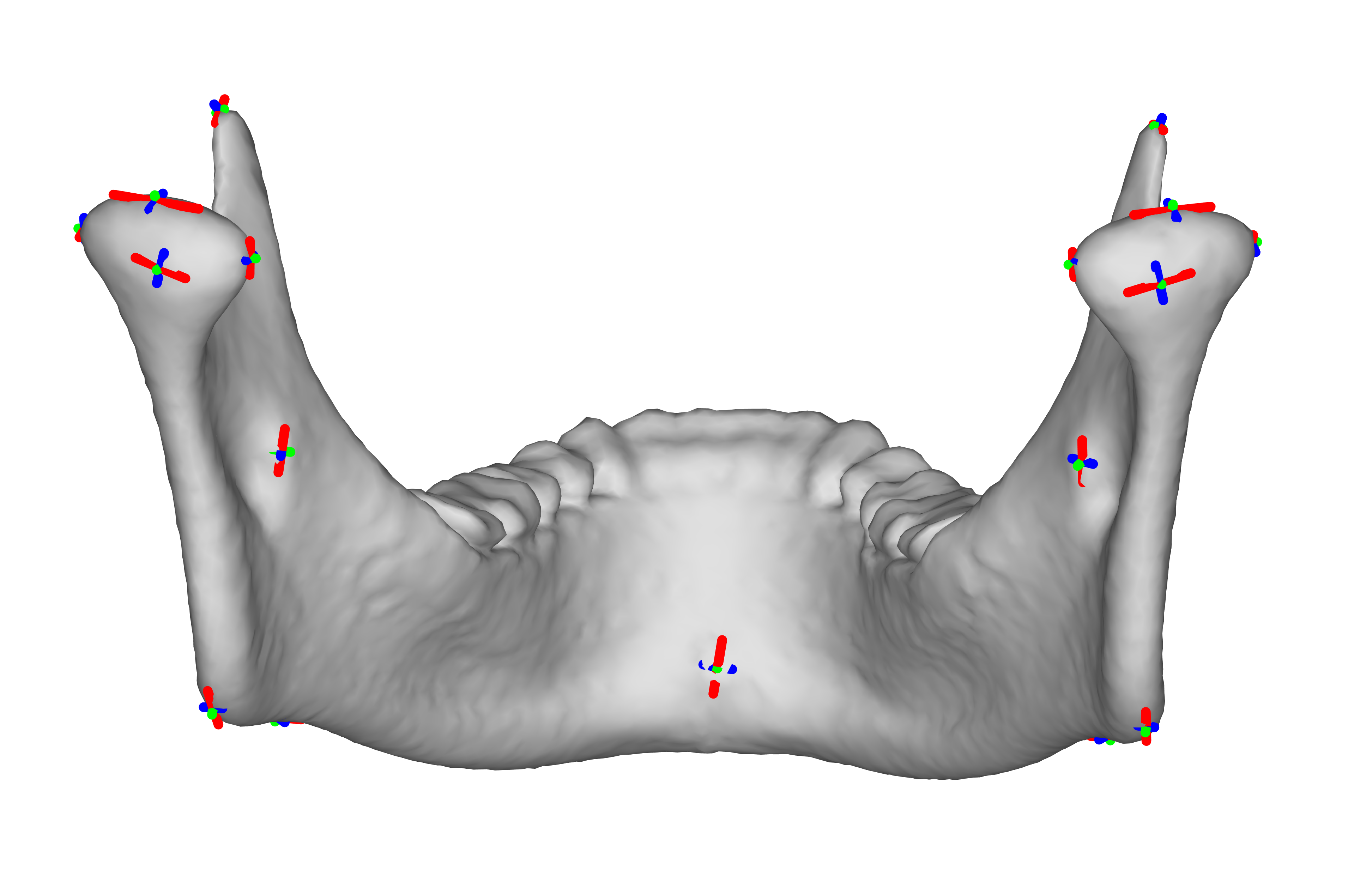


**
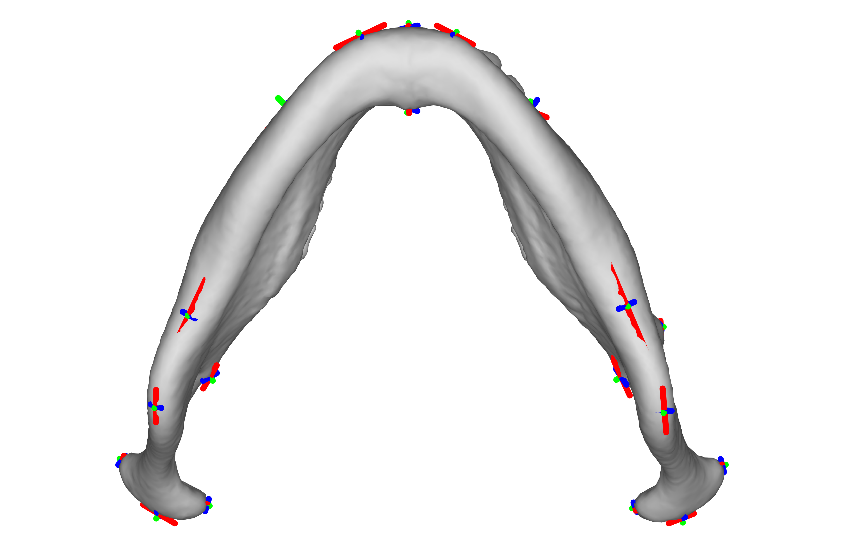
S10: Variation in error between corresponding automatic and manually identified landmarks for the operated shape sample. The illustrations provide a visual overview of the direction in which the most variation/error of landmark identification was observed. The three largest dimensions are shown for each landmark individually with red being the largest dimension, blue the second and green the third largest dimension. The table lists the magnitude of this variation in mm.**


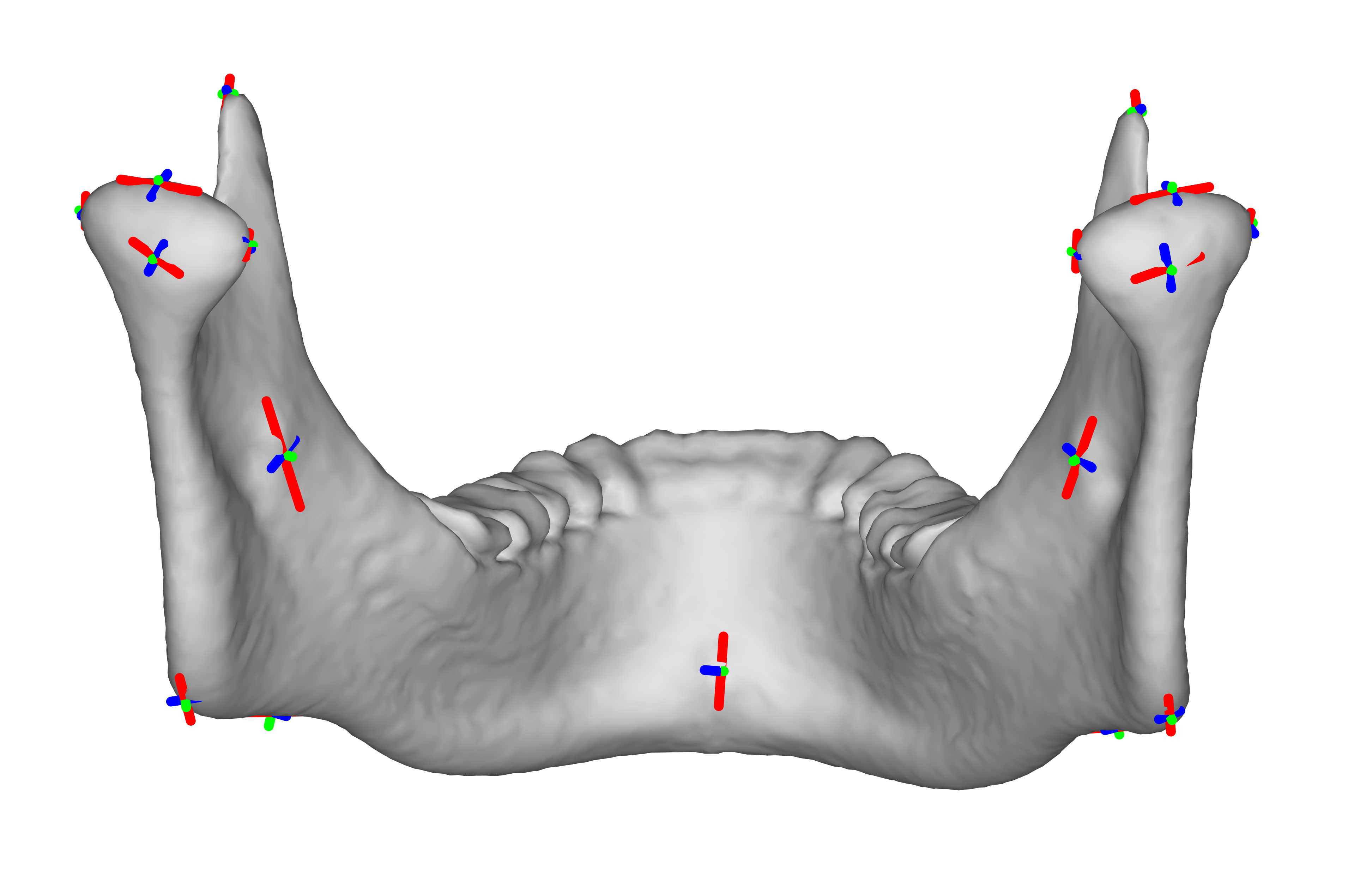

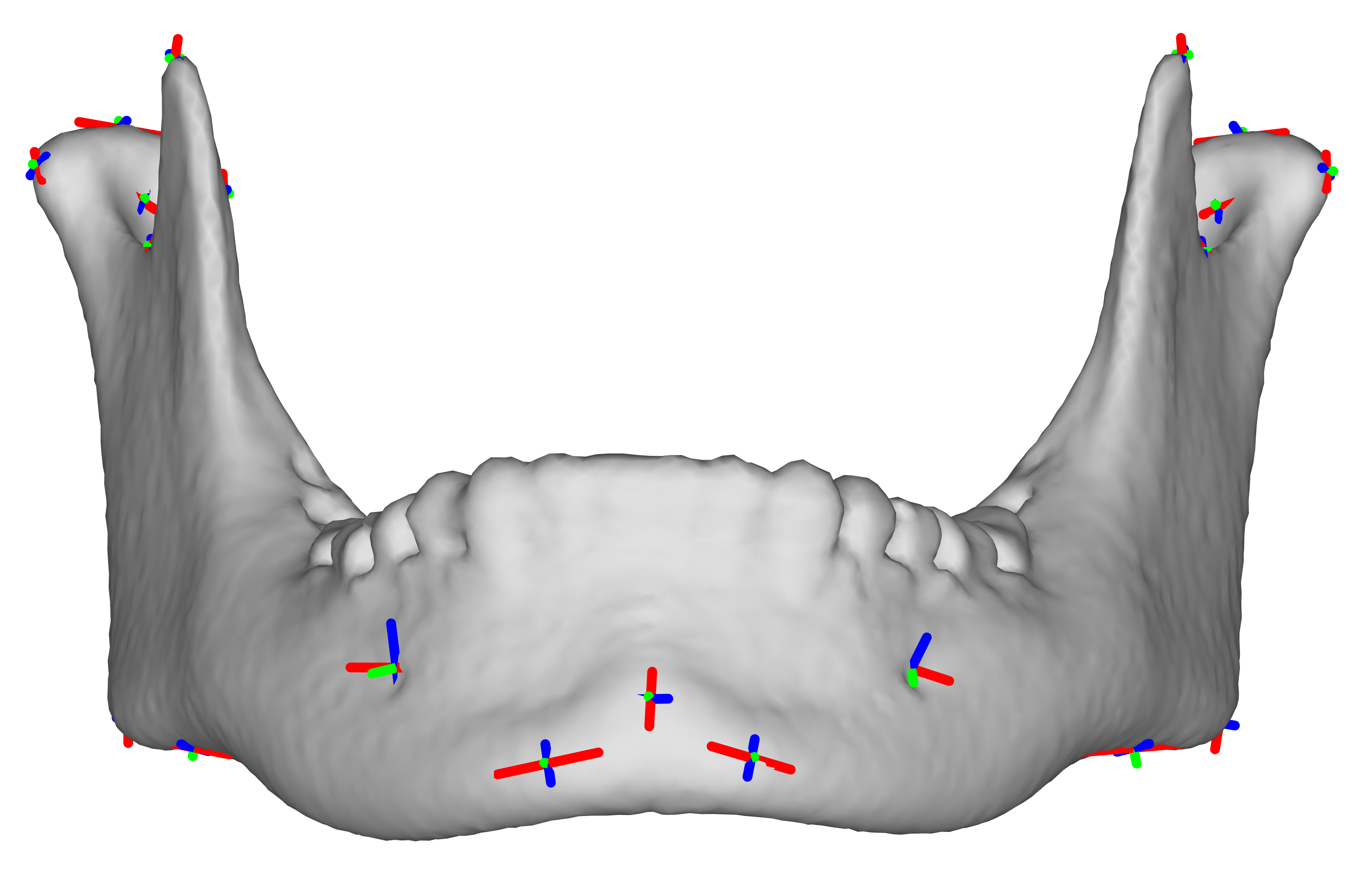


| **Landmark** | **Red** | **Blue** | **Green** |
| --- | --- | --- | --- |
| 1 | 1.95 | 0.75 | 0.42 |
| 2 | 0.90 | 0.59 | 0.26 |
| 3 | 0.86 | 0.64 | 0.30 |
| 4 | 1.78 | 1.08 | 0.50 |
| 5 | 0.74 | 0.57 | 0.27 |
| 6 | 1.18 | 0.29 | 0.23 |
| 7 | 0.90 | 0.59 | 0.27 |
| 8 | 2.49 | 0.83 | 0.45 |
| 9 | 1.94 | 0.56 | 0.30 |
| 10 | 3.80 | 0.67 | 0.38 |
| 11 | 2.84 | 1.96 | 1.54 |
| 12 | 2.49 | 0.86 | 0.39 |
| 13 | 1.25 | 0.79 | 0.41 |
| 14 | 1.69 | 0.84 | 0.47 |
| 15 | 1.95 | 0.86 | 0.34 |
| 16 | 1.81 | 1.71 | 1.17 |
| 17 | 5.17 | 0.79 | 0.71 |
| 18 | 2.59 | 0.69 | 0.42 |
| 19 | 3.59 | 1.05 | 0.54 |
| 20 | 0.88 | 0.42 | 0.29 |
| 21 | 1.45 | 0.39 | 0.25 |
| 22 | 0.87 | 0.58 | 0.35 |
| 23 | 1.41 | 0.83 | 0.41 |
| 24 | 0.80 | 0.63 | 0.33 |
| 25 | 0.62 | 0.55 | 0.31 |
| 26 | 2.07 | 0.96 | 0.33 |

**S11: SAS software syntax and comments for the mixed model analysis used to compute the contributions of mandible, observer and method to the total variance of the centroid sizes. Mixed models were fitted using PROC MIXED in SAS 9.4. using restricted maximum likelihood estimation (REML).**

SAS syntax:

proc mixed data=datacentroids  asycov method=reml alpha=**0.05** covtest;

class shapenum observer;

model centroid=;

random intercept/subject=shapenum;

random intercept/subject=observer;

ods output covparms=covparms asycov=asycov;

run;

datacentroids=dataset with (20 or 30 mandibile)*7 (observers) * 2 (methods) rows

Shapenum=unique number for each of the 20 or 30 mandibile

Observer= unique number for each of the 7 observers

Using the ‘ods output’ statement, two datasets are created, one (covparms) containing the estimates for the covariance parameters and another (asycov) containing the covariance matrix of these covariance parameters. The latter matrix is needed to calculate the 95% confidence interval for the ICC using the delta rule after a Fisher’s transformation of the ICC. The delta rule is applied using the SAS macro %NLEstimate.
